# Use of zinc deposited in deciduous teeth as a retrospective measurement of dietary zinc exposure during early development

**DOI:** 10.3389/froh.2023.1119086

**Published:** 2023-02-24

**Authors:** N. A. Wahono, L. A. Wakeling, W. Dirks, D. A. Banks, T. J. Shepherd, D. Ford, R. A. Valentine

**Affiliations:** ^1^ Pediatric Dentistry Department, Faculty of Dentistry, Universitas Indonesia, Jakarta, Indonesia; ^2^ School of Dental Science, Newcastle University, Newcastle Upon Tyne, United Kingdom; ^3^ Department of Anthropology, University of Durham, Durham, United Kingdom; ^4^ Faculty of Environment, School of Earth and Environment, University of Leeds, Leeds, United Kingdom; ^5^ Faculty of Health and Life Sciences, Northumbria University, Newcastle Upon Tyne, United Kingdom

**Keywords:** zinc, strontium, deciduous teeth, diet, food frequency questionnaire

## Abstract

**Purpose:**

We proposed that zinc (Zn) deposition in deciduous teeth would be a timed record of exposure to this essential micronutrient over very early life. We tested this hypothesis by gathering information on the maternal and child's diet during pregnancy and early infancy and measuring mineral deposition in the dentine at points during deciduous tooth development.

**Methods:**

We developed a short food frequency questionnaire (S-FFQ) to record consumption of food containing Zn during pregnancy and over the first year of life of the child in an Indonesian population. Zn, Sr and Ca were measured by laser ablation ICP-MS in a series of points across the developmental timeline in deciduous teeth extracted from 18 children undergoing the process as part of dental treatment whose mothers completed the SFFQ. Mothers and children were classified into either high Zn or low Zn groups according to calculated daily Zn intake.

**Results:**

The Zn/Sr ratio in dentine deposited over late pregnancy and 0–3 months post-partum was higher (*p* < 0.001, 2-way ANOVA; *p* < 0.05 by Holm-Sidak *post hoc* test) in the teeth of children of mothers classified as high Zn consumers (*n* = 10) than in children of mothers classified as low Zn consumers (*n* = 8).

**Conclusion:**

The S-FFQ was validated internally as adequately accurate to measure zinc intake retrospectively during pregnancy and post-partum (∼7 years prior) by virtue of the correlation with measurements of zinc in deciduous teeth. The ratio of Zn/Sr in deciduous teeth appears to be a biomarker of exposure to zinc nutrition during early development and offers promise for use as a record of prior exposure along a timeline for research studies and, potentially, to identify individuals at heightened risk of detrimental impacts of poor early life zinc nutrition on health in later life and to implement preventative interventions.

## 1. Introduction

The nutritional environment encountered during development *in utero* and early childhood has been shown in numerous studies to have potential lifelong consequences for health through a range of mechanisms including physiological impacts and epigenetic recording (1, 2). Although numerous studies have uncovered specific relationships, the need for longitudinal measurement in the context of the long human lifespan is a constraint, and much of the work has been done in animal models. Retrospective measurement of exposure in humans would be a highly valuable tool in such research. Also, some negative impacts may remain dormant and be manifest only in combination with further environmental exposures and/or as a result of ageing. Thus, it is important to uncover biomarkers of early life nutritional exposure, ideally in childhood while plasticity is still high, to intervene and re-set a better health trajectory.

The essentiality of zinc in myriad cellular and physiological functions accounts for the remarkably large component of the proteome that comprises zinc metalloproteins, which has been estimated to be 10% (3). It is beyond the scope of this paper to provide a full exposition of the myriad roles of Zn in normal physiological function and to describe in depth how zinc dyshomeostasis can affect health and increase susceptibility to or cause disease. However, there are particularly strong and well-studied associations, and recent rigorous reviews, concerning the role of zinc in immune function (4), skin health (5) and glycaemic control (6).

This high prevalence of zinc in fundamental biological function leads to the prediction that the zinc supply *in utero* will have profound effects on lifelong health. However, while there is a sizeable body of work on the value of zinc supplementation on outcomes for pregnancy and early infancy (7), little attention has been given to the potential role of maternal zinc status during pregnancy on health of the progeny in later life. Evidence of likely important impact includes a report that zinc concentration in maternal plasma during the first trimester was associated negatively with motor score and language ability at 1 year of age (8). The lack of a robust biomarker of current zinc status, despite studies over several decades that have proposed measures in hair and urine and measurement of the expression of zinc-responsive proteins such as metallothioneins, as well as measurement in plasma, introduces a level of uncertainty into studies on the effects of zinc status on health (9, 10). We propose that, because zinc is incorporated into the dental hard tissues, seemingly *via* zinc-regulated zinc transporters (11), and has been shown to influence the physical properties and thus probably resilience of enamel (12), the dietary zinc supply during development may have longer term implications for oral health. There is a need to gather robust evidence on the effects of early life nutritional exposure to zinc. To do this, the ability to determine retrospectively the level of zinc exposure of individuals during the period *in utero* would be a valuable tool. This would also facilitate the identification of individuals at risk of any detrimental impacts so that dietary remediation or other interventions to protect against specific effects of poor early zinc nutrition uncovered through future research can be recommended.

Measures of mineral deposition in teeth in relation to morphological features that identify periods of growth and weaning have a long history of use by anthropologists in the study of human life history (13). Many of these studies focused on the detection of early dietary transitions, such as weaning, using strontium (Sr) and barium (Ba) [reviewed in (14)] and exposure to the neurotoxicant lead [e.g., (15, 16)]. However, zinc deposition in enamel and dentine has also been investigated, revealing that the enamel surface is highly enriched in zinc (17, 18), which was attributed to preferential binding of zinc by matrix metalloproteinase-20 and kallikrein-4, which are active during the two-stage enamel mineralisation process of secretion and maturation. These studies dismissed the use of dentine for recording trace element incorporation because of its porous nature. However, other studies have revealed that dentine does, in fact, incorporate trace elements in a predictable way, with clear zonation and time resolution from incremental markings (15, 16, 19). This was further demonstrated using synchrotron x-ray fluorescence to map calcium, strontium and zinc at the neonatal line (NNL), which revealed increased levels of zinc in prenatal enamel and in dentine leading to the suggestion that zinc can be used to help identify the NNL (20). A more recent study that used LA-ICP-MS single line rastering of the entire crown (21) found that zinc was elevated just after birth in 65% of the sample, but variable in the rest, highlighting the need for more studies and a need to contextualise the results and take account of influencing factors. Evidence of environmental influences on metal deposition in the dentine of deciduous teeth was gathered in a pilot study on a community-based population, which used a retrospective method that accounted for water sources for both mother and infant, breastfeeding duration, formula feeding and demographic information and found correlations between early diet and trace element concentrations and timing of incorporation (19).

Given these promising preliminary results, we propose that zinc deposited in the dentine of deciduous teeth can be used as a biomarker of zinc status during early development. To address this hypothesis, we developed a food frequency questionnaire (FFQ) to measure retrospectively zinc consumption by mothers of children who presented for the extraction of deciduous teeth at the Child Integration Clinic, Dental Hospital of The Dentistry Faculty, Universitas Indonesia and we measured zinc in the dentine of these extracted teeth by laser ablation inductively-coupled plasma mass spectrometry (LA-ICP-MS) to investigate if a relationship existed.

## 2. Materials and methods

Ethical permission was obtained from the Committee of The Medical Research Ethics of the Faculty of Medicine University of Indonesia (526/UN2.F1/ETIK/2014). All participants in this study were treated based on the guidelines assigned in the Declaration of Helsinki and gave informed consent.

### 2.1. Development and validation of food frequency questionnaires (FFQ) for dietary Zn intake

A semi-quantitative FFQ to estimate dietary Zn intake in the Indonesian population was developed, focusing particularly on pregnancy and the period of infancy. In an initial phase, participants filled out an online questionnaire to report their recollection of all foods consumed in the previous 24 h (Q-24 h) to gather information on foods commonly consumed in Indonesia. The food items gathered from the Q-24 h were used in the development of a FFQ to be used to estimate habitual Zn intake over a longer period (LFFQ). As this study focused on Zn intake, food items not captured through the Q-24 h but known to be good sources of Zn were added. The LFFQ comprised 82 food items.

A series of food photographs were produced to enable participants to estimate their usual food portion and were based on the recommendations of a previous study (22). Each food was presented as four portion sizes comprising 25%, 50%, 100% and 125% of a portion commonly consumed or portion on the package label of commercial products. Portions were measured out using an electrical scale (TANITA digital food scale). The amount of Zn in each food was obtained from USDA Nutrient Database for Standard Reference (United States Department of Agriculture 2013; https://data.nal.usda.gov/dataset/usda-national-nutrient-database-standard-reference-legacy-release), the Indonesian food database Nutrisurvey 2007 (http://www.nutrisurvey.de), or from previous studies (23, 24). A plate or bowl containing the food was arranged together with a spoon and fork on each side. Food was photographed on a white background using a digital camera with a macro lens (Nikon 3100D) and photographs were printed at a size of 4 cm × 8 cm. In parallel, a shorter version of the FFQ (S-FFQ), which comprised fewer food items (28 items), was developed with the aim of reducing the required time for completion and thus pressure on the interviewer and participant during the clinic visit. To develop the S-FFQ, the number of food items was reduced by focusing on Zn-rich foods, such as red meat, offal, avocado, broccoli, spinach, grouping vegetables with lower Zn content, such as cabbage, carrot and lettuce, into a category of “other vegetables” and excluding items that were found to be rarely or never consumed by this population, such as brown rice, veal and pork. The L-FFQ and S-FFQ were compared with one and other and with a 3-day food record (Q3-d).

Both the L-FFQ and the S-FFQ consisted of five sections, which were: (1) personal information about child and parents, which included name, date of birth, birth weight, parents' educational background and occupation; (2) prenatal and birth history; (3) post-natal history, including feeding in the first six months, weaning age and foods, and consumption of food supplements; (4) retrospective record of foods consumed during pregnancy; (5) retrospective record of foods consumed by the child during infancy (from weaning up to age one year old); and (6) record of foods consumed by the child at the point of sampling. The S-FFQ is included as supplementary information.

### 2.2. Calculation of current dietary Zn intake and intake during pregnancy and infancy

Children who attended the Child Integration Clinic, Dental Hospital of The Dentistry Faculty, Universitas Indonesia, from July-August 2014, for the removal of deciduous teeth to address dental health issues were eligible for this study. The inclusion criteria were: (1) the child and mother declared their willingness to participate in the study by signing a consent form explained to them previously; (2) the crown structure of the extracted tooth/teeth was still intact. Dental health personnel carried out the clinical assessment and tooth extraction following standard dental procedures.

Mothers were interviewed at the single visit when extraction was carried out to complete the overarching study questionnaire and the S-FFQ for themselves (retrospective recall for the period during pregnancy) and their children (over the period from weaning to one year old and current). Before completing the FFQ for foods consumed during pregnancy, the mothers were asked about their health during pregnancy, morning sickness and any food cravings. They were also asked to describe all foods they consumed on a daily (or frequent) basis, from breakfast to dinner. Portion size was estimated by the participants from the food photographs, and frequency of consumption was recorded. Daily Zn intake from the food source was calculated from these parameters and all intakes were summed to calculate total average daily Zn intake. Any information given by the participants was treated as confidential. They repeated the FFQ, but this time describing the food consumed by their child during infancy and again a FFQ for their child at their current age.

### 2.3. Zn and Sr distribution in dentine of human primary teeth

Extracted teeth were rinsed in PBS (137 mM NaCl, 2.7 mM KCl, 4.3 mM Na_2_HPO_4_.7H_2_O, 1.4 mM KH_2_PO_4_, pH 7.3) to remove blood and debris then placed immediately in RNAlater® Stabilization Solution (Invitrogen) at 4°C. Teeth were stored at 4°C overnight then transferred to −20°C storage in the Oral Biology Laboratory, Faculty of Dentistry, Universitas Indonesia. Transfer to the Oral Biology Laboratory, School of Dental Sciences, Newcastle University, was in RNALater.

**Figure 1:**
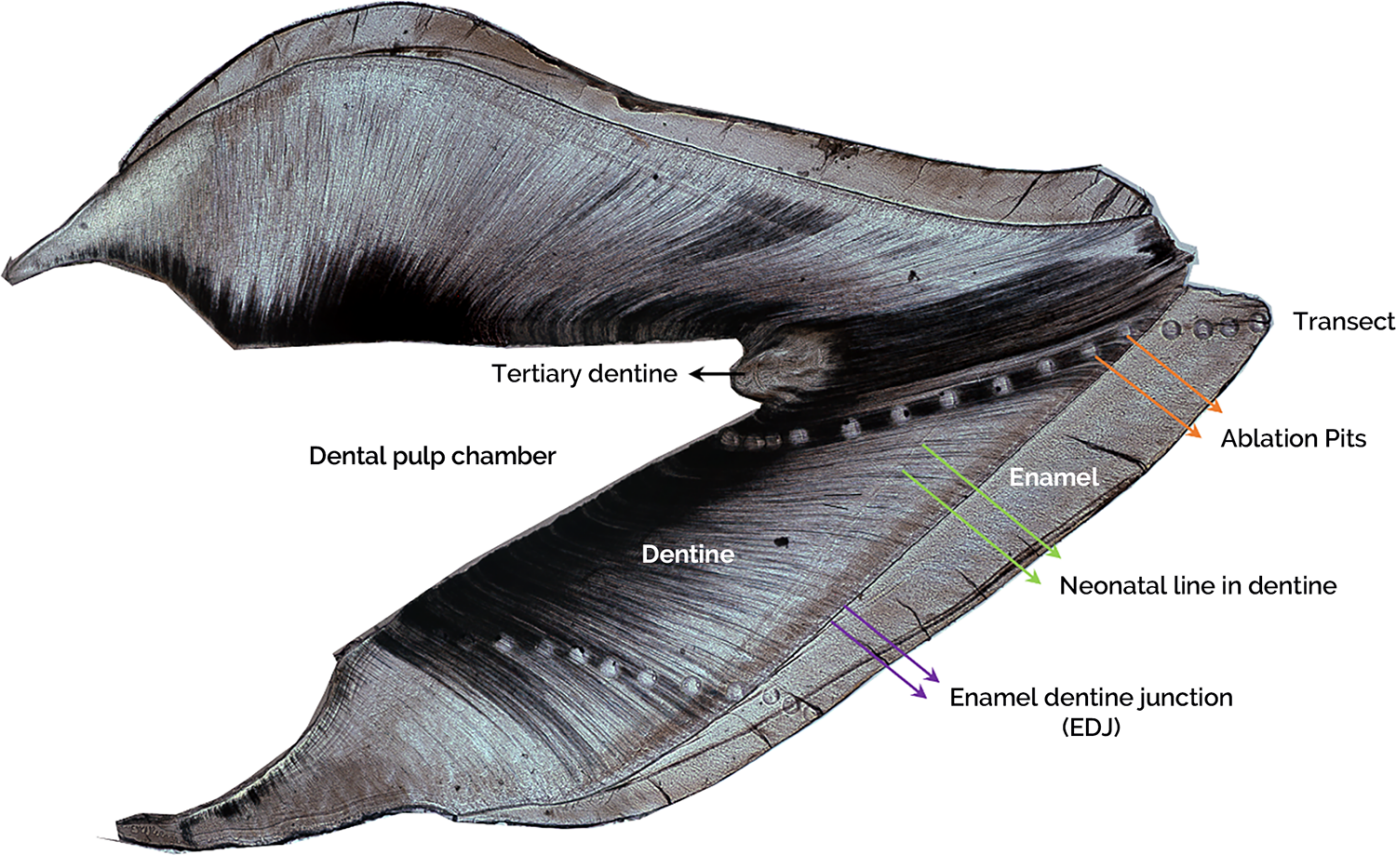
A representative image of a longitudinal ground tooth section of an incisor. Labelling shows the enamel, enamel dentine junction (EDJ, purple arrows), dentine, neonatal line (NL, green arrows), ablation pits in the transect (orange arrows), dental pulp chamber (DPC), and tertiary dentine (black arrow).

Teeth were sectioned to 200 µm thickness using a using a low-speed saw. Sections were prepared from the middle one third in the mesial-distal direction for incisors and at least two cusp tips were included in the buccal-lingual direction for molars. Trace elements including Ca, the major cation of dentine, were measured by Laser Ablation Inductively Coupled Mass Spectrometry (LA-ICP-MS) at the Faculty of Environment, School of Earth and Environment, University of Leeds based on a published protocol (15). Briefly, a Geolas 193 nm ArF excimer laser coupled to an Agilent 750°C ICP-mass spectrometer was used to vaporise dentine tissue from a series of 100 µm diameter pits (constant energy density of 10 J/cm^2^; pulse rate of 5 Hz). Ablation pit transects, which crossed the neonatal line, were generated along the dentinal tubules from the enamel dentine junction (EDJ) towards the dentine-pulp chamber (DPC) (Figure 1). The laser ablation process was captured using a video camera with visible light sources integrated into the optical array to be displayed and monitored continuously on a computer screen. The specification and operating conditions of the laser and mass spectrometer are summarised in Table 1.

**Table 1:** Laser and ICP-MS specifications and operating conditions.

Laser model	GeoLas ArF excimer
Laser wavelength	193 nm
Laser energy	10 J/cm^2^
Laser pulse rate	5 Hz
Pulses per ablation analysis	200
Beam diameter	100 μm
ICP-MS model	Agilent 750°C quadrupole MS
Scanning mode	Peak jumping
Background acquisition time	∼20 s
Signal acquisition time	∼40 s
Dwell times	10 ms ^44^Ca, ^66^Zn, ^88^Sr

Reference material NIST SRM Glass 610 was analysed before and after each analytical session and used for instrument calibration. This also permitted cross referencing to replicate analyses of NIST SRM Glasses 612 and 614 to determine the instrument performance and within-run standard errors. The isotopes chosen for elemental measurement (44Ca, 66Zn, 88Sr) were checked and proven free from isobaric interferences. Measured ion intensities were processed offline using SILLS, a software programme specifically written for signal integration of laboratory laser systems by Murray Allan (University of Leeds) and later modified by Dimitri Meier and Marcel Guillong (Die Eidgenossische Technische, Zurich). The data were then presented as elemental ion intensities (cps) relative to Ca ion intensities (cps) as Zn/Ca and Sr/Ca cps ratios. In the absence of international reference standards for dentine (bio-apatite), cps ratios permitted relative variation in Zn and Sr in tooth dentine to be studied without the need for absolute concentrations.

### 2.4. Determination of tooth age from pregnancy up to twelve months of age

After the LA-ICP-MS procedure, all ground sections were observed under a polarized light microscope (Olympus BX51). The images were captured (x4 objective) and processed using a Q-Imaging Micropublisher 3.3 RTV camera and Improvision Openlab 5.0.2 image software. The tooth sections were then further prepared by polishing down the section into 80–100 µm thickness, and individually mounted on a microscope slide. Each ablation pit was identified, and the diameter was measured in micrometer (µm) units. The distance between ablation pits were also determined. The daily secretion rate of dentine (DSR) in each area was calculated by measuring the distance between Von Ebner's lines (x40 objective). All measurements were carried out using ImageJ software. The period of time covered by each pit and gap was determined by dividing the width of each by the DSR. Using the neonatal line (NNL; indicated on Figure 1 *NNL is more commonly used in histology literature*.) in dentine as the zero point, the age range (days) for each ablation pit was determined, taking the median value as the age point (days) of the ablation pit. Age points for ablation pits laid in dentine from the NNL to the enamel dentine junction (EDJ) are stated as negative values.

## 3. Results

### 3.1. Development and validation of a food frequency questionnaire (FFQ) for dietary Zn intake

The S-FFQ was validated as a tool to measure Zn intake in adult women by comparing the daily Zn intake recorded using the S-FFQs with a 3-day diet record (3DR) in six volunteers. A Bland-Altman plot was used to evaluate the limits of agreement between the S-FFQ and 3DR. The differences between the two assessments were within the standard deviation of the mean difference (0.19 for 3DR minus S-FFQ), which was 3.97 mg/d for the upper limit and −3.59 mg/d for the lower limit. Thus, the S-FFQ was considered adequately accurate and used in the study to estimate the daily Zn intake of the participants.

The L-FFQ and S-FFQ as tools to measure Zn intake in children were compared through completion by thirteen mothers of children who attended Al-Hidayah kindergarten, Bekasi, West Java, Indonesia. On the first day, participants were interviewed to complete the S-FFQ to calculate the daily Zn intake of their children in the previous month. A follow-up interview was carried out three days later to complete the L-FFQ for the same month. Statistical analysis confirmed a significant and positive strong correlation in dietary Zn intake measured using the two FFQs (Spearman analysis, *r* = 0.997, *p* = 0.01). Bland-Altman analysis showed that the daily Zn intake measured using the S-FFQ was in good agreement with the intake calculated using the L-FFQ. The differences in daily Zn intake measured using the L-FFQ and S-FFQ were within the standard deviation of the mean difference (−0.33 for L-FFQ minus S-FFQ), which was 2.6 mg/d for the upper limit and −3.26 mg/d for the lower limit. It was thus deemed that the S-FFQ was an acceptable replacement for the L-FFQ and that its use in the study would not compromise the accuracy of data obtained on dietary Zn intake in the child participants.

### 3.2. Estimation of dietary Zn intake during pregnancy and infancy

Eighteen children paired with their mothers were eligible and recruited in this study. Characteristics of participants, including age, medical history, socioeconomic level, prenatal and postnatal history, are shown in Table 2. The dental hospital is located at central Jakarta, and the majority of the participants originated from the local area.

**Table 2:** Characteristics of the study participants (*n* = 18).

General Information		*n* (%)
Child's Gender	Male	8 (40.00)
	Female	10 (50.00)
Child's age (years) (mean ± SD)	7.94 ± 1.25
Special needs	0 (0.00)
Medical history	Illness	0 (0.00)
	Allergies	5 (26.32)
Socioeconomic level
Father's education	below high school	1 (5.00)
	high school	12 (60.00)
	higher education	5 (25.00)
Mother's education	below high school	2 (10.00)
	high school	11 (55.00)
	higher education	5 (25.00)
Family income (Indonesian Rupiah)	<1,000,000	2 (10.00)
	1,000,000–<3,000,000	8 (40.00)
	3,000,000 < 5,000,000	5 (25.00)
	≤5,000,000	3 (15.00)
Prenatal and birth history
Mother's age during pregnancy [years (mean ± SD)]	27.94 ± 5.07
Hyperemesis	7 (35.00)
Hospitalized	1 (5.00)
Drugs/Food Supplements/Vitamins		18 (90.00)
Delivery time	pre-term	3 (15.00)
	at-term	16 (80.00)
Delivery method	vaginal birth	14 (70.00)
	caesarean section	4 (20.00)
Birthweight (kg) (mean ± SD)	3.17 ± 0.57
First six-months of feeding
Exclusively breastfed	7 (50.00)
Breastfed + early weaned	4 (22.4)
Breastfed + formula-fed	3 (16.68)
Breastfed + formula-fed + early weaned	3 (16.68)
Formula-fed	1 (5.56)
Weaning history	
Weaning age (months) (mean ± SD)	4.86 ± 2.29
Daily Zn intake (mg) (mean ± SD)
Pregnancy	11.79 (7.31)
Infancy	5.96 (2.89)
At point of sampling	7.54 (3.70)

The daily Zn intake of the mother during pregnancy and the child during infancy were calculated using the S-FFQ. The estimated average requirement (EAR) for Zn in the mixed or refined vegetarian group defined by the IZiNCG [International Zinc Nutrition Consultative Group (IZiNCG) et al., 2004] was used to classify participants into low and high Zn intake groups, taking the EAR as the boundary between groups. The EAR for pregnant women is 8 mg/d and for children aged 1–3 years old the EAR is 3 mg/d. Eight mothers were in the low Zn intake (LZM) group (intake <8 mg/d); ten mothers were in the high Zn intake (HZM) group (intake >8 mg/d). Two children were in the LZC group (intake <3 mg/d); sixteen were in the HZC group (intake >3 mg/d).

The foods consumed most frequently during pregnancy, infancy, and at the point of sampling were rice, cereals and noodles (grouped). The food consumed least frequently was offal. Plant-based protein made the highest total contribution to daily Zn intake in mothers, followed by (in rank order) dairy and its products, rice, cereals and noodles (grouped), red meat and its products, eggs, beans, seeds and nuts (grouped), Zn-rich vegetables and fruits (grouped), fish and seafood, white meat and its products, and offal.

During infancy, the greatest contribution to daily Zn intake was from dairy products, followed by (in rank order) eggs, plant-based protein, rice, cereals and noodles (grouped), white meat and its products, Zn-rich vegetables and fruits (grouped), red meat and its products, offal, fish and seafood, beans, seeds and nuts (grouped).

At the time of sampling, the greatest contribution to daily Zn intake in children was from rice, cereals and noodles (grouped), followed by (in rank order) dairy products, red meat and its products, plant-based proteins, white meats and its products, eggs, Zn-rich vegetables and fruits (grouped), beans, seeds and nuts (grouped), fish and seafood, and offal.

**Figure 2:**
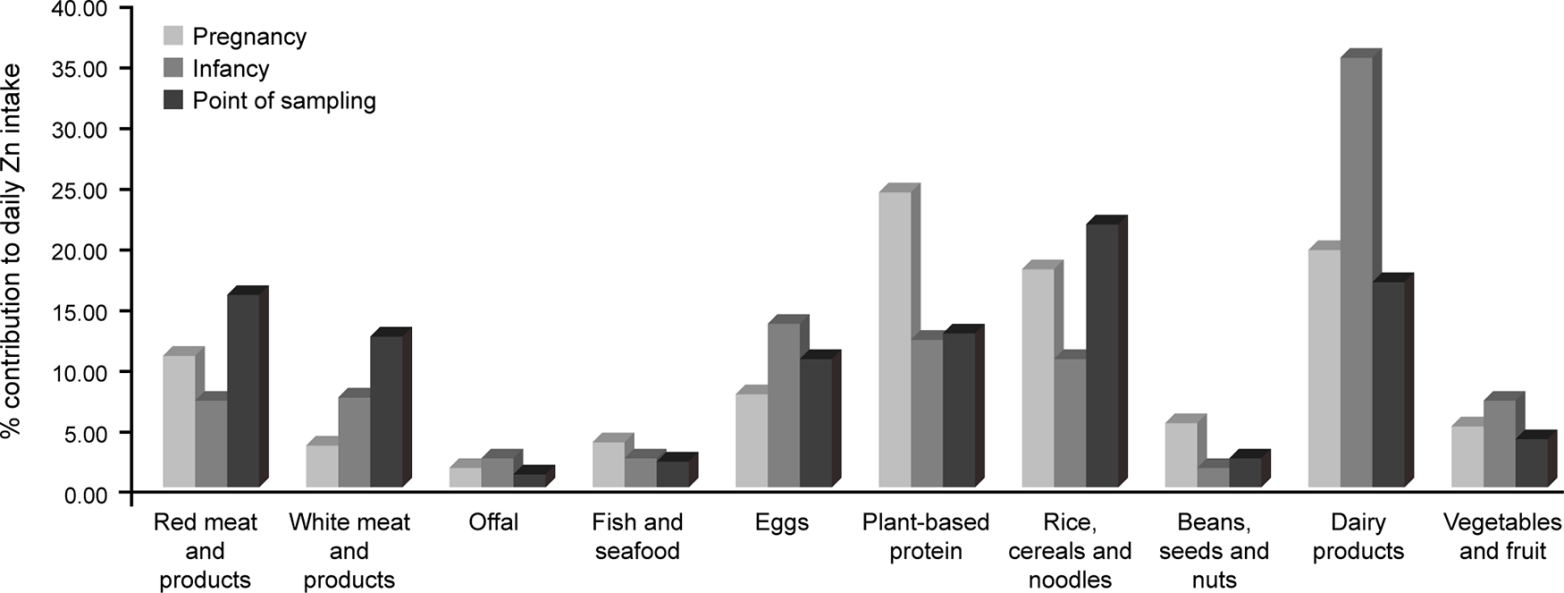
The contribution of different food groups to daily Zn intake of the study sample, measured using the S-FFQ, during pregnancy and – for children – infancy and the point of sampling.

Figure 2 shows the relative contribution of these foods for each group/time point.

### 3.3. Distribution of Zn and Sr in the dentine of human primary teeth

^66^Zn, ^40^Ca, and ^88^Sr were measured in one primary tooth from each of the study participants, which consisted of 14 incisors and 4 molars. Sr distribution and content was measured for comparison with Zn distribution and content, and we posited that it would be unrelated to developmental exposure to dietary Zn. Results presented relative to Ca ion intensity use a multiplication factor of 10,000 for Zn and 1,000 for Sr.

**Figure 3:**
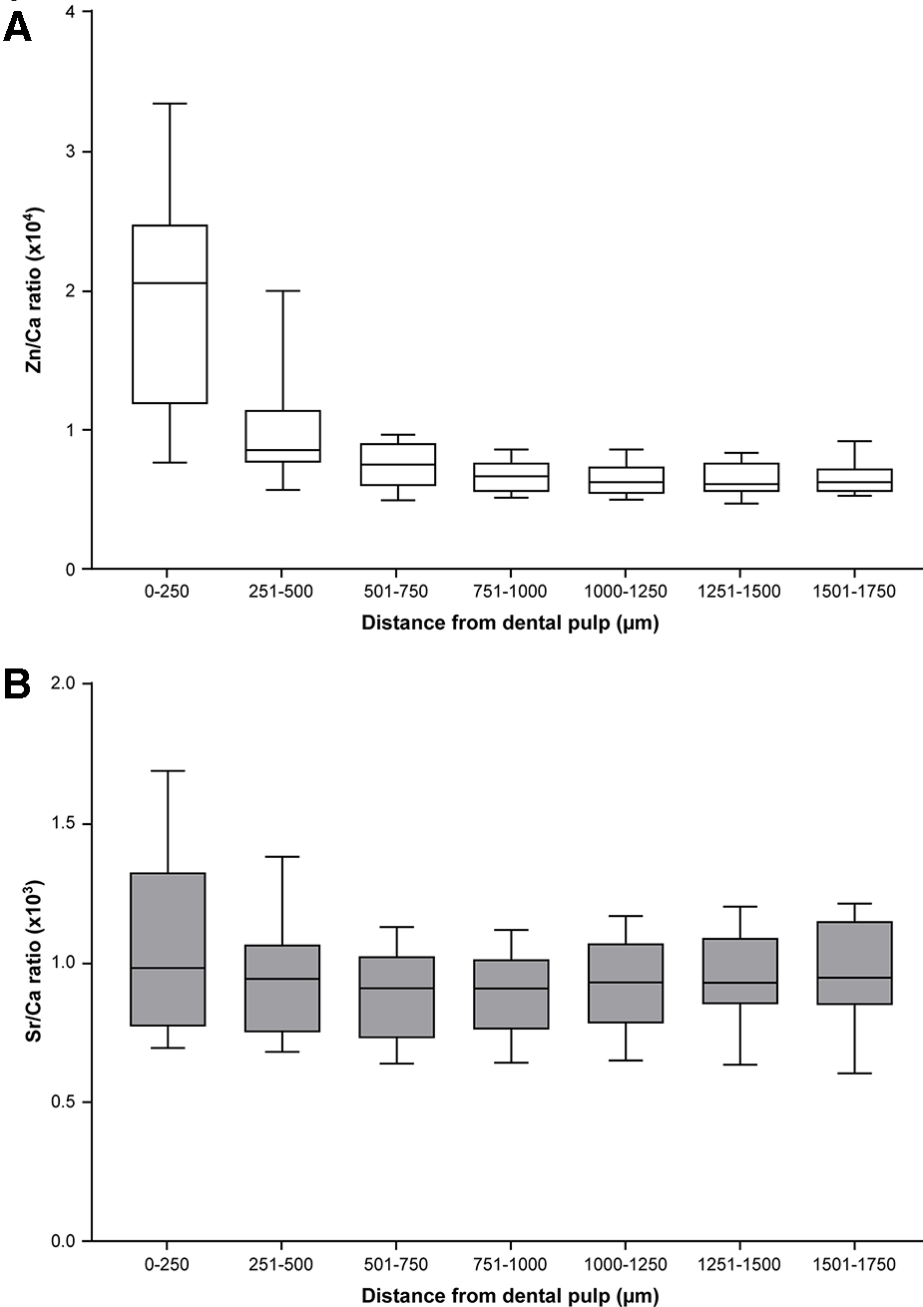
Distribution of Zn and Sr between the pulp and EDJ in deciduous teeth (median and 25th–75th percentile with whiskers extending from smallest to largest value; *n* = 18). (**A**) The Zn/Ca ratio differed in ablation pits according to the distance from the pulp to the EDJ (Friedman test, *p* < 0.0001). The ratio was significantly higher in ablation pits closest to the pulp [a,b *p* < 0.05, c,d *p* < 0.01 (Dunn's post-hoc test)]. (**B**) The Sr/Ca ratio in ablation pits also differed according to distance from the pulp to the EDJ (Friedman test, *p* < 0.0001) and was significantly lower in ablation pits in the region 501–750μm from the DPC compared with the region closer to and more distant from the DPC (*p < 0.05, Dunn's post-hoc test).

A previous study (15) found a sharp increase in Zn in ablation pits within approximately 200 µm of the DPC, and hypothesized this was associated with the formation of secondary dentine. In this area, the dentinal tubule density and diameter increase and accommodate more odontoblast cells involved in secondary dentine formation. Consistent with the findings of this earlier study, we found that the Zn/Ca ratio was significantly higher in the region within 500 μm of the DPC compared with other points (Figure 3A). The Sr/Ca ratio was greater closer to the pulp and the EDJ compared with the area 501–750 μm from the DPC (Figure 3B). These results suggest differences in the mechanisms and their regulation for incorporation of Zn and Sr in dental hard tissue.

To align ablation pits, and thus the corresponding measurements of Zn and Sr, with time points during development, we excluded measurements up to 500 μm from the DPC, on the basis that it was likely to constitute a region of secondary dentine formation. Thus, for incisors the median (minimum-maximum) time points covered were 111 (47–220) days before birth to 321 (277–517) days after birth, and for molars the median (minimum-maximum) time points covered 87 (40–127) days before birth to 492 (427–559) days after birth.

### 3.4. Zn and Sr distribution in dentine from developmental time points of pregnancy up to twelve months of age

**Figure 4:**
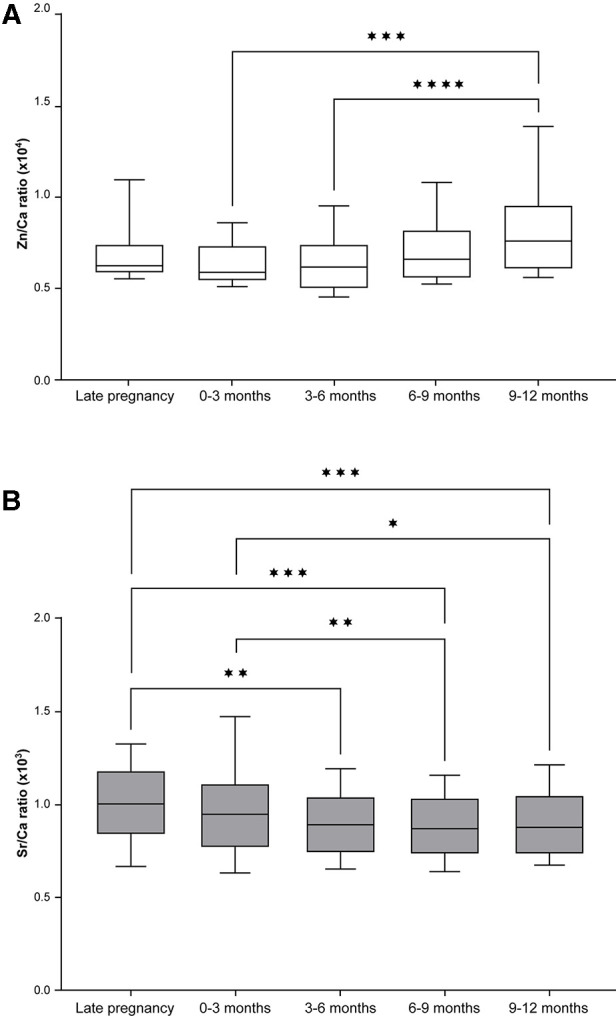
Zn and Sr distribution across different points in time during the development of deciduous teeth (median and 25th–75th percentile with whiskers extending from smallest to largest value; *n* = 18). (**A**) The Zn/Ca ratio differed significantly across time points (Friedman test, *p* < 0.001); ***p < 0.001, ****p < 0.0001 (Dunn's *post hoc* test). (**B**) The Sr/Ca ratio also differed significantly across time points (Friedman Test, *p* < 0.001); *p < 0.05, **p < 0.01, ***p < 0.001 (Dunn's *post hoc* test).

The Zn/Ca ratio remained fairly constant between late pregnancy and 0–3 months but then increased up to the latest sampling point of 9–12 months, differing significantly between 0 and 3 months and 9–12 months and between 3 and 6 months and 9–12 months (Figure 4A). In contrast there was a significant decrease in the Sr/Ca ratio across all time intervals measured (Figure 4B).

### 3.5. The impact of Zn nutrition on Zn and Sr distribution in dentine

**Figure 5:**
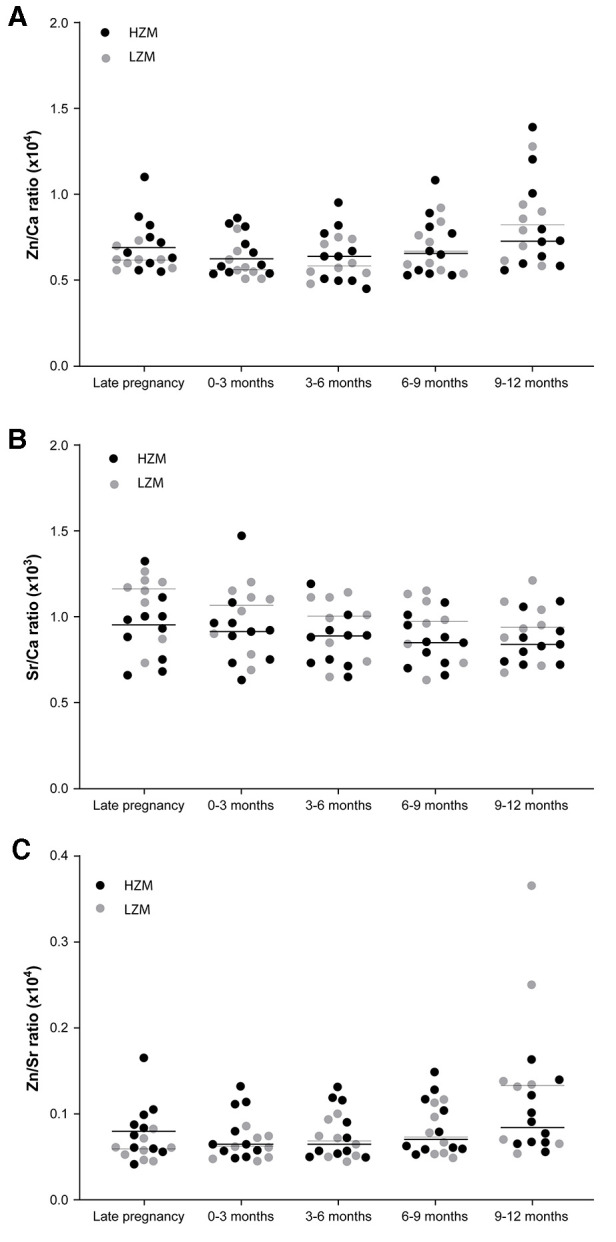
Ratios of Zn, Sr and Ca across different points in time during the development of deciduous teeth shown as scatter plots with data points coded for mothers classified as consumers of a high zinc (HZM; *n* = 10) or low zinc (LZM; *n* = 8) diet during pregnancy. Medians are shown as horizontal bars. (**A**) Data expressed as Zn/Ca ratios. (**B**) Data expressed as Sr/Ca ratios. (**C**) Data expressed as Zn/Sr ratios; *p* < 0.001, 2-way ANOVA; *p < 0.05 for HZM vs. LZM (Holm-Sidak *post hoc* test).

Separation of the data on Zn/Ca and Sr/Ca ratios in dentine according to maternal classification into high or low zinc intake groups (HZM (*n* = 10) and LZM (*n* = 8), respectively) revealed a different trend for Zn compared with Sr that was consistent with our hypothesis that higher Zn intake during development *in utero* and early infancy would be recorded as higher Zn levels in dentine but that Sr would not show this relationship (Figure 5). The median Zn/Ca ratio for the HZM group from ablation pits corresponding with all time points sampled was numerically higher than for the LZM group, although there were no statistically-significant differences (Figure 7A). In contrast, Sr/Ca ratios did not show this trend but showed an opposite trend, which we did not predict, with ratios from ablation pits corresponding with all time points sampled being numerically higher in LZM than in HZM groups, though again not differing significantly (Figure 5B). However, when data were expressed as Zn/Sr ratios there was a significant difference according to dietary zinc group (*p* < 0.001, 2-way ANOVA; Figure 5C). The Zn/Sr ratio in ablation pits corresponding to late pregnancy and 0–3 months post-partum was higher in the teeth of children of HZ mothers compared with LZ mothers (*p* = 0.024, Holm-Sidak *post hoc* test).

## 4. Discussion

The most significant finding of this study was that dietary intake of Zn during pregnancy measured using a food frequency questionnaire developed for the purpose of measuring this retrospectively in the mothers of children of ages at which the primary dentition was still present (median 7.94 years) was correlated with the quantity of Zn as a ratio of Sr deposited during tooth development. The internal consistency of this finding indicates that retrospective recall of the diet eaten by the mothers in the sample during pregnancy was an adequate basis on which to determine Zn intake over this period and that the S-FFQ developed was a sufficiently accurate tool to capture this information. External validation of the S-FFQ *per se* was demonstrated by its use to measure current dietary Zn intake in adult women living in Indonesia through comparison with 3-day food records in a sample of 6 women.

Given the much broader importance to lifelong health of the child of the maternal diet more generally during pregnancy, knowledge that a short food frequency questionnaire administered retrospectively can be informative with regard to measuring intake of a specific nutrient is useful information to other researchers wishing to survey diet during pregnancy in a similar retrospective manner. However, an important caveat is that extension of this principle to other cultures cannot be assumed. Cultural dietary norms may introduce more uncertainly and inaccuracy in other populations, thus similar tools would require suitable validation in other contexts.

Studies on zinc intake and zinc status of mothers, infants and children in Indonesia indicate that inadequate zinc intake and zinc deficiency are prevalent. For example, in West Java, 25% of mothers and 17% of their infants (2.4–10.5 months) were found to be zinc-deficient (25), and a recent study found that 43.2% of a sample of children in a rural village in Indonesia had inadequate zinc intake and the majority of these had low serum zinc concentration (26). A sample of women living in the coastal area of Makassar all had low serum zinc concentration (<66 microg/100 ml) 4–6 weeks after giving birth and 21% had had zinc intake of less that 80% RDI (average 15.9 mg/d) (27). The calculated average daily zinc intake in mothers in our sample (11.8 mg) was lower than that of this sample from Makassar (15.9 mg), which indicates that children in our sample from urban areas around Jakarta will have been exposed to sub-optimal zinc nutrition during early life. The apparent prevalence of inadequate zinc intake in Indonesian mothers and children mean that future studies on the impacts of inadequate early zinc nutrition on the lifelong health of the child are important. A retrospective measurement of this, as we show to be afforded by measurement in the deciduous dentition, will – compared with longitudinal studies - expediate outcomes of research on this topic.

Our discovery that the Zn/Sr ratio in deciduous teeth correlated with maternal Zn intake during late pregnancy (median 111 days or 87 days before giving birth for incisors and molars, respectively) and 0–3 months post-partum, when most infants were breastfed, should be agnostic of population and dietary culture. Thus, we have uncovered a biomarker of early developmental exposure to Zn nutrition that could be applied independently of dietary records in other studies and in other populations to uncover new information about the effect of early life Zn nutrition on later health outcomes.

Seven of the 18 children (3 of high Zn mothers and 4 of low Zn mothers) were breastfed exclusively up to the age of 6 months. The significant effect of the maternal diet on the Zn/Sr ratio was still evident in the areas mineralised from late pregnancy to 3–6 months in this group. This is consistent with `the mineral record being reflective of indirect dietary exposure through breast milk rather than only a consequence of receiving foods more similar to the maternal diet in the full group of 18 children. However, this significant effect was lost when all time points (also 6–9 and 9–12 months) were included in the model. A likely explanation is a combination of a reduction in the power of the statistical analysis combined with a reduced influence of the maternal diet as breastfeeding was replaced with other foods.

Evidence that Zn exposure *in utero* can affect susceptibility to features of the metabolic syndrome, such as cardiovascular disease (28), and the association of Zn status with type 2 diabetes mellitus (6), points to a hypothesis that Zn status in very early life may influence susceptibility to type 2 diabetes in later life. We posit an interrogation of this hypothesis as one of many potential future studies using retrospective measures of zinc exposure during very early life made using laser ablation ICP-MS in deciduous teeth.

We measured Sr primarily because some data suggest that the Sr/Ca ratio can be used as an indicator of the period of breastfeeding. We observed that the median Sr/Ca ratio decreased across the time intervals sampled. It was argued, and demonstrated in a small sample of human deciduous teeth, that the ratio should decline after birth if the infant is breastfed because of a greater activating effect of the mammary gland than the placenta on Ca transfer but increase if the infant is bottle-fed (29). Extending this argument predicts that weaning should coincide with an increase in the ratio. In our study, the ratio decreased after birth in 14 of the 18 teeth, commensurate with this prediction and these previous observations. However, the predicted relationship with bottle-feeding was not observed. Two of the infants were bottle-fed immediately or within 2 weeks of birth and both showed a decline in Sr/Ca after birth. We observed no increase in Sr/Ca in any of the teeth after weaning (reported by mothers as ranging from 2 to 8 months; mode 6 months) nor on the reported point at which table foods were introduced, which for 5 of the infants was within the 12 month sampling period. Thus, although the pattern of change in the Sr/Ca ratio was consistent with the observations reported previously our observations do not support the hypothesis that this is due to an activating effect of breast milk on Ca transfer. The measurement of Sr in deciduous teeth along with other elements, as in this study, may be useful in future research in the context of its purported but debated cariostatic properties (30). While some *in vitro* studies have demonstrated that Sr – particularly in combination with Fl - can promote caries rehardening - e.g., (31) - epidemiological data are confounded by the co-presence in the water supply of other trace elements with possible similar properties. Use of the trace element profile of the deciduous dentition alongside records of caries may deconvolute some of these interactions and shed more light on the role Sr can play in protection against dental caries.

## 5. Conclusion

Maternal dietary Zn intake correlated with the ratio of Zn/Sr deposited in the developing tooth over the period of late pregnancy and early infancy. This measure is a promising tool to record exposure to Zn during this period of development for use in research and also, potentially, to identify individuals at heightened risk of detrimental impacts of poor early life Zn nutrition on health in later life and to implement preventative interventions.

## Data Availability

The raw data supporting the conclusions of this article will be made available by the authors, without undue reservation.
