# Supplementary material for: Use of zinc deposited in deciduous teeth as a retrospective measurement of dietary zinc exposure during early development
Source: Front Oral Health. 2023 Feb 24;4:1119086. doi: 10.3389/froh.2023.1119086 (PMC9998501; doi:10.3389/froh.2023.1119086)

**General Questionnaire**

**EXPLORING THE LEVELS AND DISTRIBUTION OF ZINC IN THE DECIDUOUS TOOTH AS A TOOL TO RECORD EARLY LIFE EXPOSURE TO ZINC NUTRITION**

**Personal Information Code:**

| **CHILD** |  | **FAMILY** |  |
| --- | --- | --- | --- |
| Name |  | Father’s Name |  |
| Gender *(tick one of the answer)* | 🞎 MALE / 🞎 FEMALE | Education Level | School/College/University |
| Date of Birth |  | Occupation |  |
| Place of Birth |  | Mother’s Name |  |
| Nationality |  | Education Level | School/College/University |
| School |  | Occupation |  |
| Body Weight | __________ kg | Number of Children in family | ____________person(s), and the child is number _______ |
| Body height | __________cm |  |  |
| Childhood Illnesses  *(tick one of the answer)* | 🞎 YES  🞎 NO  if yes, please to mention it below:  ___________________ | Home Address |  |
| Disabilities  *(tick one of the answer)* | 🞎 YES  🞎 NO  if yes, please to mention it below:  ___________________ | Phone / Mobile Phone |  |
|  |  | Annual Household Expense/month *(tick one of the answer)* | 🞎 < £10,000  🞎 £10,000 - £20,000  🞎 £20,000 - £40,000  🞎 >£50,000 |

- **PRENATAL AND BIRTH HISTORY -**

| 1. Mother age during pregnancy | ______________y.o. |
| --- | --- |
| 1. Morning sickness   *(tick one of the answer)* | 🞎 YES  🞎 NO |
| 1. Complication during pregnancy (admitted to hospital)   *(tick one of the answer)* | 🞎 YES  🞎 NO  If YES, details______________________________________ |
| 1. Medication/supplement   (eg. Folic acid) during pregnancy  *(tick one of the answer)* | 🞎 YES  🞎 NO  If YES, please mention the type of medication/supplement that was taken: __________________________________________  and duration ________________week(s)/ month(s) |
| 1. Gestational Age   *(tick one of the answer)* | 🞎 Pre- term, __________ week(s)/ month(s)  🞎 At- term, __________ week(s)/ month(s) |
| 1. Labour and Delivery Method   *(tick one of the answer)*  🞎 Vaginal Birth  🞎 Vaginal Birth with intervention (forceps/ventouse)  🞎 Caesarian Section | 1. Baby weight at birth ________ kg |

- **POST NATAL HISTORY -**

| **1.** | **Feeding Pattern**  *(tick one of the answer)* | 🞎 **Exclusive Breastfeeding,**  (please continue to question number 2)  🞎 **Mixed Breastfeeding and Formula**  (please continue to question number 3)  🞎 **Formula**  (please continue to question number 4) |
| --- | --- | --- |

| **2.** | **Exclusive Breastfeeding :** From _____week/month to _______week/month |
| --- | --- |

| **3.** | **Mixed Breastfeeding and Formula**   1. **Breastfeeding :** From _____week/month to _______week/month 2. **Formula :** From _____week/month to _______week/month |
| --- | --- |

| **4.** | **Formula :** From _____week/month to _______week/month |
| --- | --- |

***-* WEANING HISTORY *-***

| **1.** | **Age of the baby when you started weaning** | _______month/year |
| --- | --- | --- |
| **2.** | **The first food for your child** | 🞎 **Homemade baby food,**  that was ____________________  🞎 **Commercially available baby food,**    that was ____________________ |
| **3.** | **Age of the child when he/she was fully weaned (eating solid food)** | _______month/year |

**-Vitamin/Supplement for the Child-**

| **Do you routinely give a supplement to your child?**  *(tick one of the answer)* | 🞎 YES  🞎 NO  If YES, please mention the name or content of the supplement :  **______________________________** |
| --- | --- |
| **Do you routinely give a vitamin to your child?**  *(tick one of the answer)* | 🞎 **YES** *(you can choose more than one option below)*  🞎 Multivitamin + Mineral  🞎 Certain Vitamin, details :__________________  **🞎 NO** |

**Shortened -FFQ**

| **HOW OFTEN DOES YOU/YOUR CHILD EAT DURING PREGNANCY / INFANCY / AT-PRESENT:** *(cross one of answer)* | | | | | | | | | | | | | | | | | | |
| --- | --- | --- | --- | --- | --- | --- | --- | --- | --- | --- | --- | --- | --- | --- | --- | --- | --- | --- |
|  | **NEVER** | **EVERY MONTH** | | | **EVERY WEEK** | | | | | | **EVERY DAY** | | | **PORTION** | | | | |
|  |  | 1 | 2 | 3 | 1 | 2 | 3 | 4 | 5 | 6 | 1 | 2 | 3 | A | B | C | D |  |
| **Protein source** |  |  |  |  |  |  |  |  |  |  |  |  |  |  |  |  |  |  |
| Beef /lamb |  |  |  |  |  |  |  |  |  |  |  |  |  |  |  |  |  |  |
| Beef sausage/meatball |  |  |  |  |  |  |  |  |  |  |  |  |  |  |  |  |  |  |
| Chicken |  |  |  |  |  |  |  |  |  |  |  |  |  |  |  |  |  |  |
| Chicken nugget |  |  |  |  |  |  |  |  |  |  |  |  |  |  |  |  |  |  |
| Eggs |  |  |  |  |  |  |  |  |  |  |  |  |  | A half egg | 1 egg | 2 eggs | 3 eggs |  |
| Cow/Chicken's offal |  |  |  |  |  |  |  |  |  |  |  |  |  |  |  |  |  |  |
| Salt-water fish |  |  |  |  |  |  |  |  |  |  |  |  |  |  |  |  |  |  |
| Fresh water fish |  |  |  |  |  |  |  |  |  |  |  |  |  |  |  |  |  |  |
| Seafood |  |  |  |  |  |  |  |  |  |  |  |  |  |  |  |  |  |  |
| Tofu |  |  |  |  |  |  |  |  |  |  |  |  |  |  |  |  |  |  |
| Tempeh |  |  |  |  |  |  |  |  |  |  |  |  |  |  |  |  |  |  |
| **Rice and cereals** |  |  |  |  |  |  |  |  |  |  |  |  |  |  |  |  |  |  |
| Rice |  |  |  |  |  |  |  |  |  |  |  |  |  |  |  |  |  |  |
| White bread |  |  |  |  |  |  |  |  |  |  |  |  |  |  |  |  |  |  |
| Pasta/noodle |  |  |  |  |  |  |  |  |  |  |  |  |  |  |  |  |  |  |
| Breakfast cereal |  |  |  |  |  |  |  |  |  |  |  |  |  |  |  |  |  |  |
|  |  |  |  |  |  |  |  |  |  |  |  |  |  |  |  |  |  |  |
|  |  |  |  |  |  |  |  |  |  |  |  |  |  |  |  |  |  |  |
|  | **NEVER** | **EVERY MONTH** | | | **EVERY WEEK** | | | | | | **EVERY DAY** | | | **PORTION** | | | | |
|  |  | 1 | 2 | 3 | 1 | 2 | 3 | 4 | 5 | 6 | 1 | 2 | 3 | A | B | C | D |  |
| **Beans and nuts** |  |  |  |  |  |  |  |  |  |  |  |  |  |  |  |  |  |  |
| Long beans |  |  |  |  |  |  |  |  |  |  |  |  |  |  |  |  |  |  |
| Mung beans |  |  |  |  |  |  |  |  |  |  |  |  |  |  |  |  |  |  |
| Peanuts |  |  |  |  |  |  |  |  |  |  |  |  |  |  |  |  |  |  |
|  |  |  |  |  |  |  |  |  |  |  |  |  |  |  |  |  |  |  |
| **Vegetables & fruits** |  |  |  |  |  |  |  |  |  |  |  |  |  |  |  |  |  |  |
| Corn |  |  |  |  |  |  |  |  |  |  |  |  |  |  |  |  |  |  |
| Potato |  |  |  |  |  |  |  |  |  |  |  |  |  |  |  |  |  |  |
| Spinach |  |  |  |  |  |  |  |  |  |  |  |  |  |  |  |  |  |  |
| Mushroom |  |  |  |  |  |  |  |  |  |  |  |  |  |  |  |  |  |  |
| Broccoli |  |  |  |  |  |  |  |  |  |  |  |  |  |  |  |  |  |  |
| Other vegetables (carrot, cabbage, etc) |  |  |  |  |  |  |  |  |  |  |  |  |  |  |  |  |  |  |
| Avocado |  |  |  |  |  |  |  |  |  |  |  |  |  |  |  |  |  |  |
|  |  |  |  |  |  |  |  |  |  |  |  |  |  |  |  |  |  |  |
| **Dairy** |  |  |  |  |  |  |  |  |  |  |  |  |  |  |  |  |  |  |
| Milk |  |  |  |  |  |  |  |  |  |  |  |  |  | <250ml | 250 | >250ml |  |  |
| Cheese |  |  |  |  |  |  |  |  |  |  |  |  |  |  |  |  |  |  |
| Yogurt |  |  |  |  |  |  |  |  |  |  |  |  |  |  |  |  |  |  |

**Zn and Phytate content of foods listed in shortened FFQ**

|  |  | **Zn content**  **(mg/100 g)** | **Phyate**  **(mg/100 g)** | **Portion 1**  **(g)** | **Portion 2**  **(g)** | **Portion 3**  **(g)** | **Portion 4**  **(g)** |
| --- | --- | --- | --- | --- | --- | --- | --- |
| 1 | Beef/lamb | 5.00 | 0.00 | 20.00 | 40.00 | 80.00 | 100.00 |
| 2 | Beef sausage/meatball | 2.16 | 0.00 | 30.00 | 45.00 | 75.00 | 90.00 |
| 3 | Chicken | 2.00 | 0.00 | 30.00 | 60.00 | 120.00 | 150.00 |
| 4 | Chicken nugget | 2.00 | 0.00 | 15.00 | 30.00 | 60.00 | 90.00 |
| 5 | Eggs | 0.8/pcs | 0.00 | 0.80 | 1.60 | 2.40 | 3.20 |
| 6 | Offal | 4.4 | 0.00 | 40.00 | 80.00 | 120.00 | 160.00 |
| 7 | Salt-water fish | 0.4 | 0.00 | 25.00 | 50.00 | 100.00 | 125.00 |
| 8 | Fresh-water fish | 0.8 | 0.00 | 25.00 | 50.00 | 100.00 | 125.00 |
| 9 | Seafood | 2.3 | 0.00 | 12.00 | 25.00 | 37.50 | 50.00 |
| 10 | Tofu | 2.3 | 314.00 | 50.00 | 100.00 | 150.00 | 200.00 |
| 11 | Tempe | 1.25 | 89.5 | 10.00 | 40.00 | 60.00 | 80.00 |
| 12 | Rice | 0.35 | 33.60 | 50.00 | 100.00 | 150.00 | 200.00 |
| 13 | White Bread | 1.00 | 0.00 | 12.50 | 25.00 | 50.00 | 75.00 |
| 14 | Pasta/noodle | 0.43 | 12.47 | 30.00 | 60.00 | 90.00 | 130.00 |
| 15 | Breakfast cereal | 2.3 | 0.00 | 10.00 | 20.00 | 30.00 | 40.00 |
| 16 | Long beans | 0.67 | 25.24 | 25.00 | 50.00 | 75.00 | 100.00 |
| 17 | Mung beans | 0.9 | 121.00 | 50.00 | 75.00 | 100.00 | 125.00 |
| 18 | Peanuts | 3.27 | 2.95 | 25.00 | 50.00 | 100.00 | 125.00 |
| 19 | Corn | 1.00 | 152.00 | 37.5 | 75.00 | 112.5 | 150.00 |
| 20 | Potato | 0.3 | 60.90 | 25.00 | 50.00 | 75.00 | 100.00 |
| 21 | Spinach | 0.7 | 8.91 | 30.00 | 60.00 | 120.00 |  |
| 22 | Mushroom | 0.5 | 0.00 | 35.00 | 70.00 | 105.00 | 140.00 |
| 23 | Broccoli | 0.6 | 0.00 | 40.00 | 80.00 | 120.00 | 160.00 |
| 24 | Other vegetables (carrot, cabbage, etc) | 0.25 | 0.00 | 25.00 | 50.00 | 100.00 | 125.00 |
| 25 | Avocado | 0.45 | 0.00 | 50.00 | 75.00 | 150.00 | 200.00 |
| 26 | Milk | 1 mg/250ml | 0.00 | 125.00 | 250.00 | >250 |  |
| 27 | Cheese | 3.75 | 0.00 | 30.00 | 60.00 | 90.00 | 120.00 |
| 28 | Yogurt |  | 0.00 | 100ml | 125ml | 250ml |  |

**Appendix B Food-portion size photographs**

1.
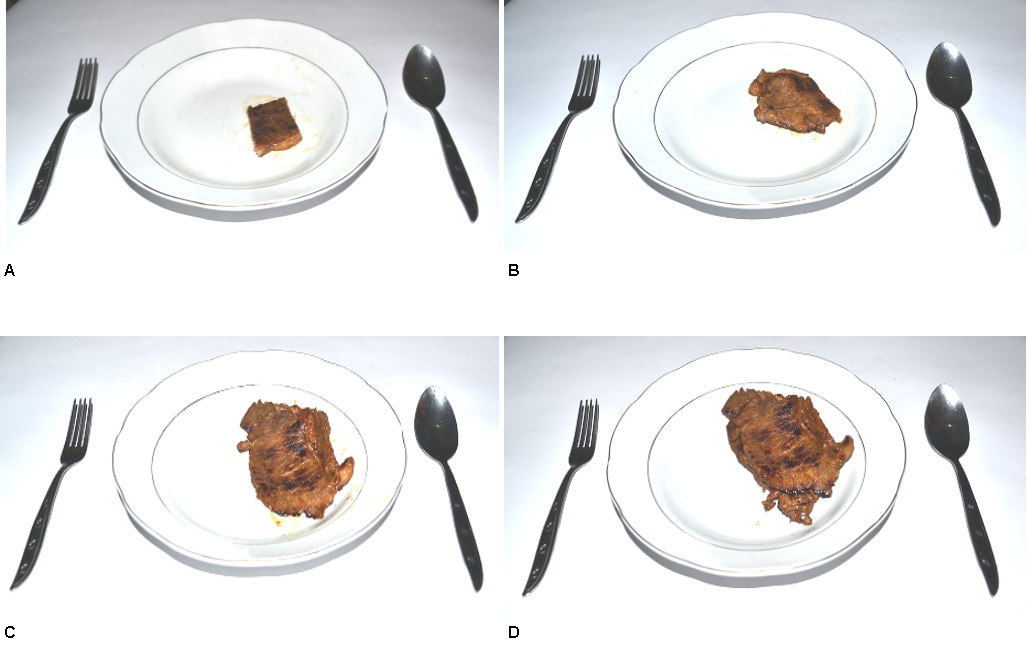
Red Meat
2.
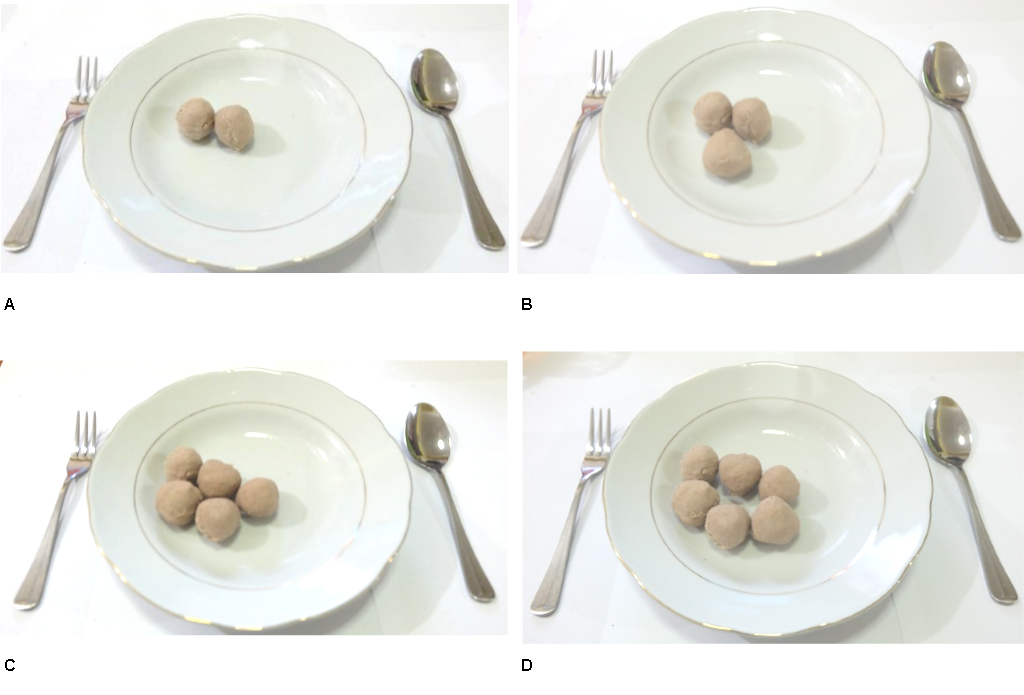
Meatballs
3.
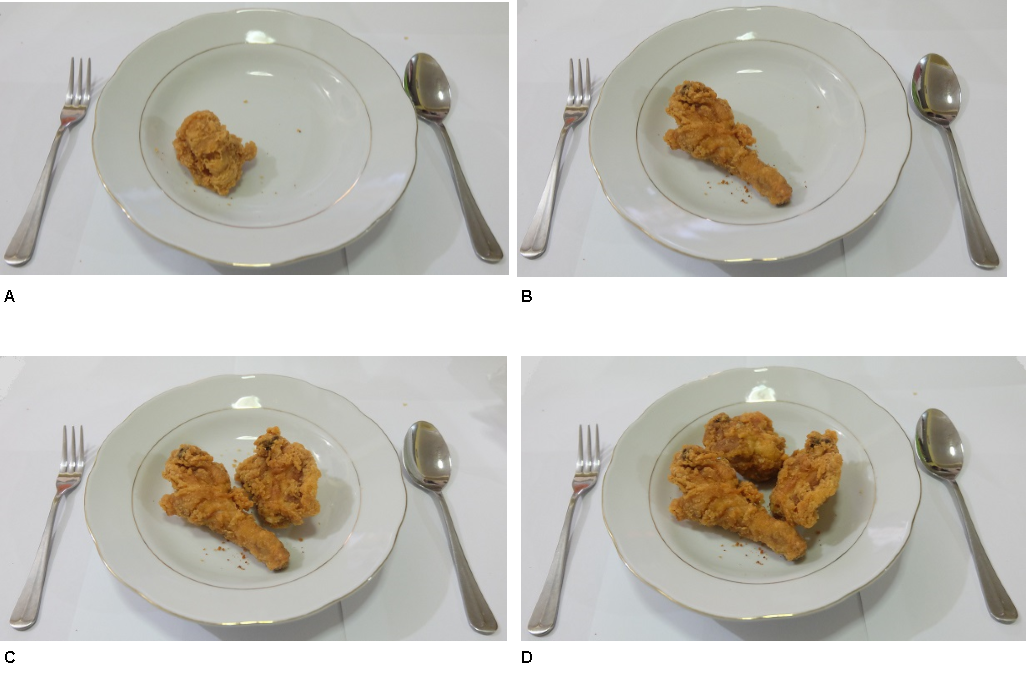
Chicken
4. Chicken Nugget


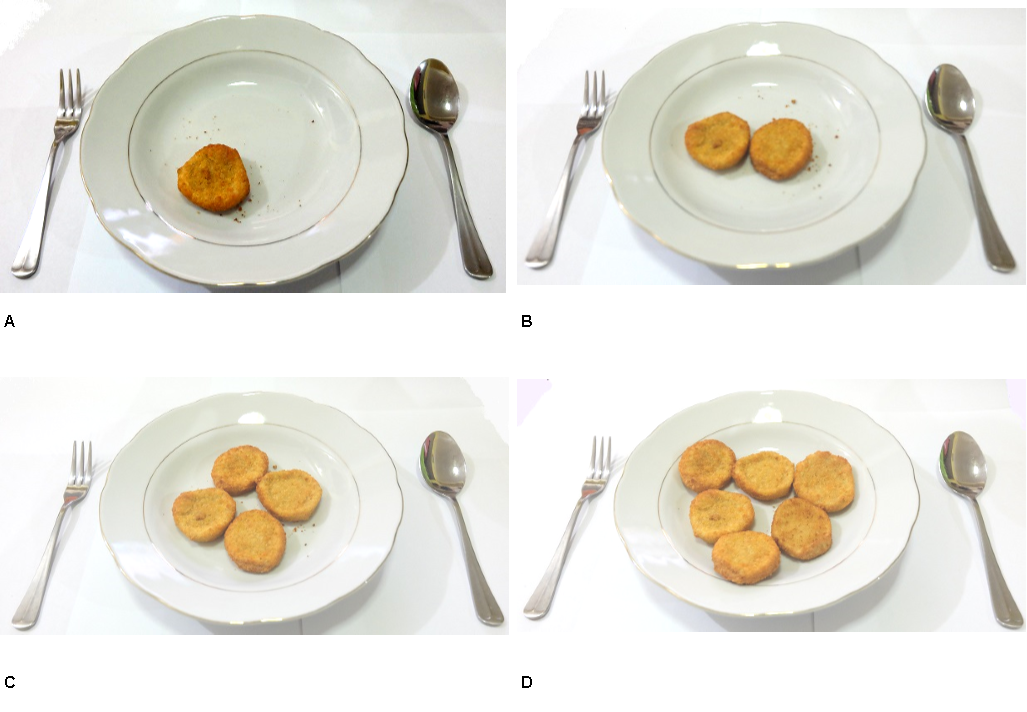


1.
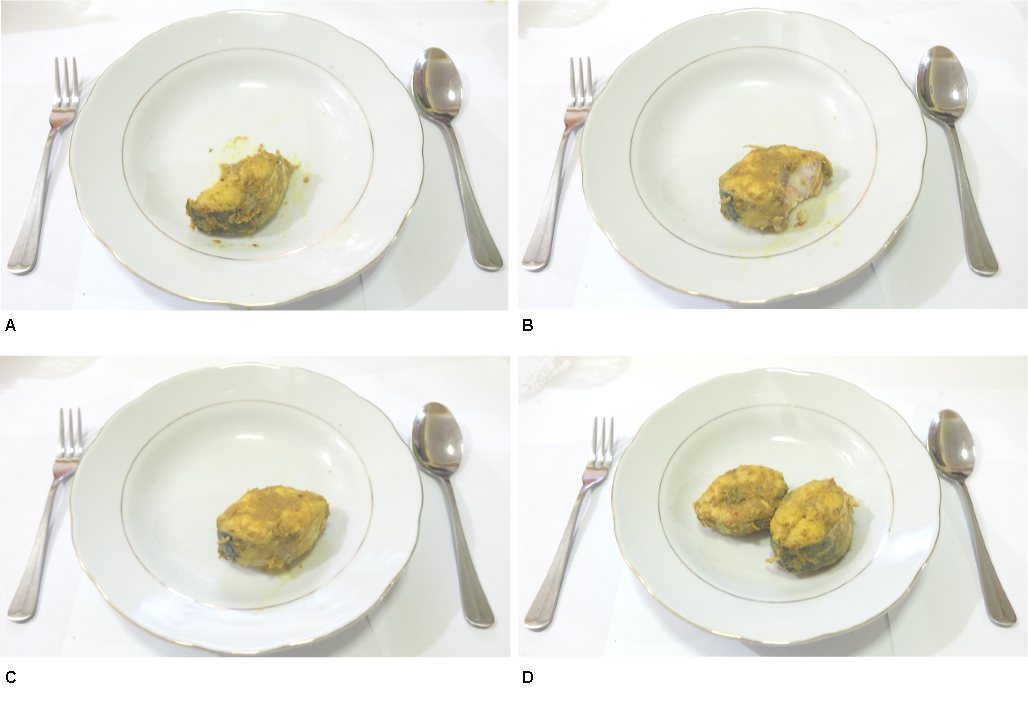
Salt-water fish
2. Fresh water fish


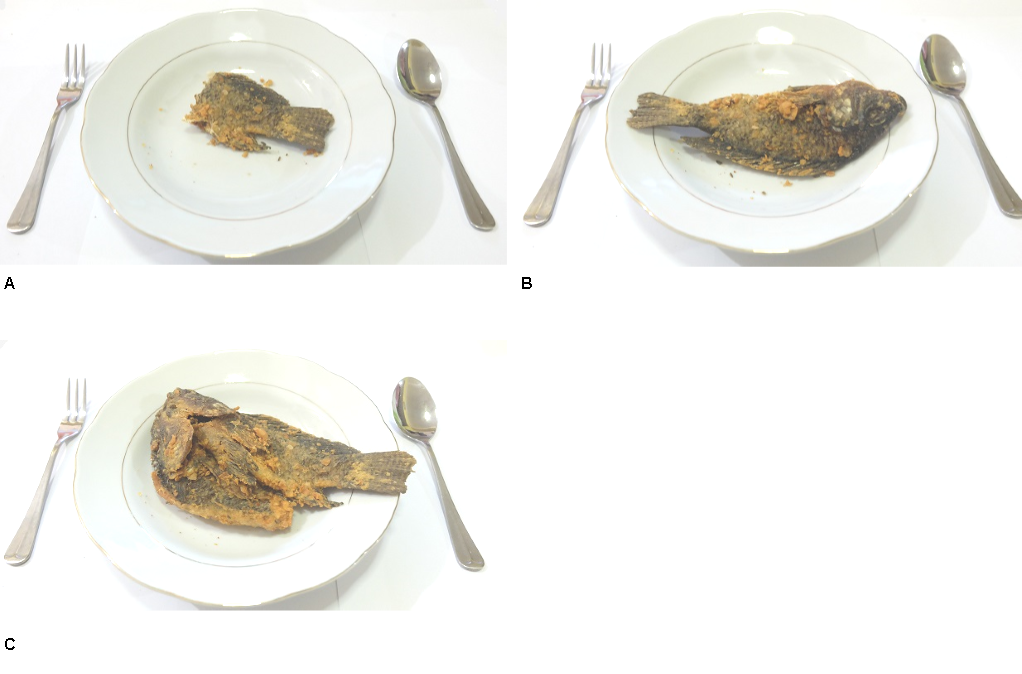


1. Seafood


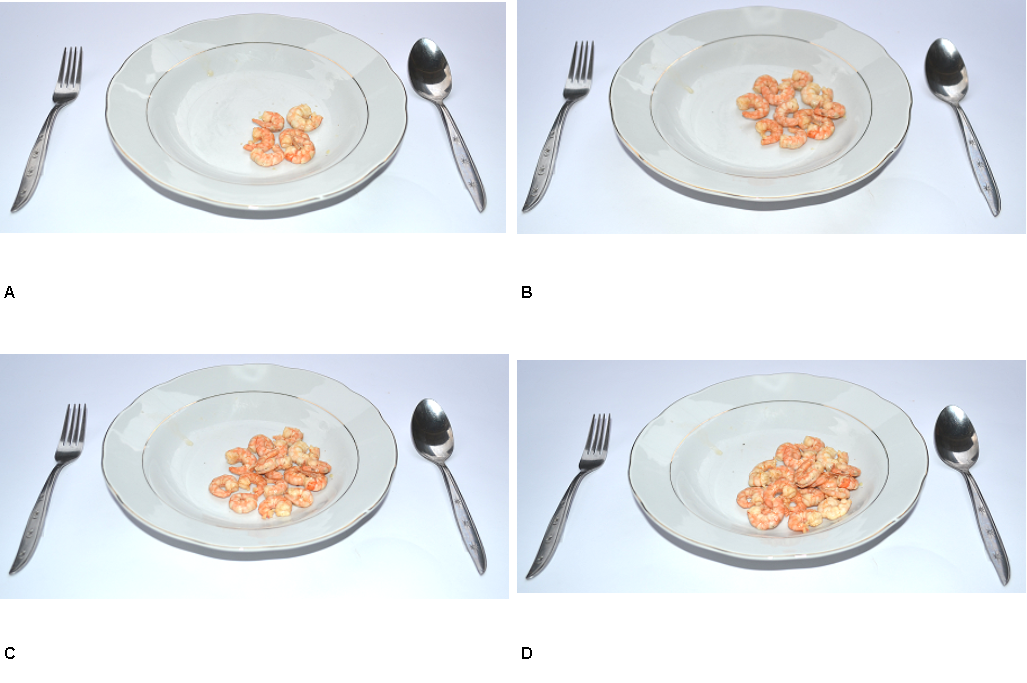


1. Tofu


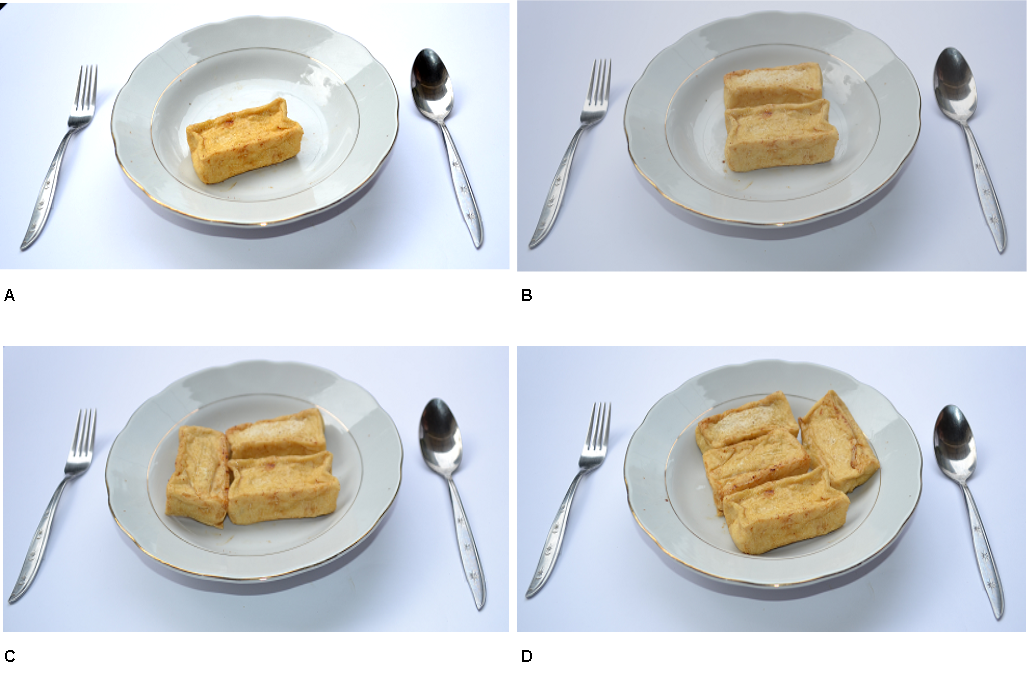


1. Tempeh


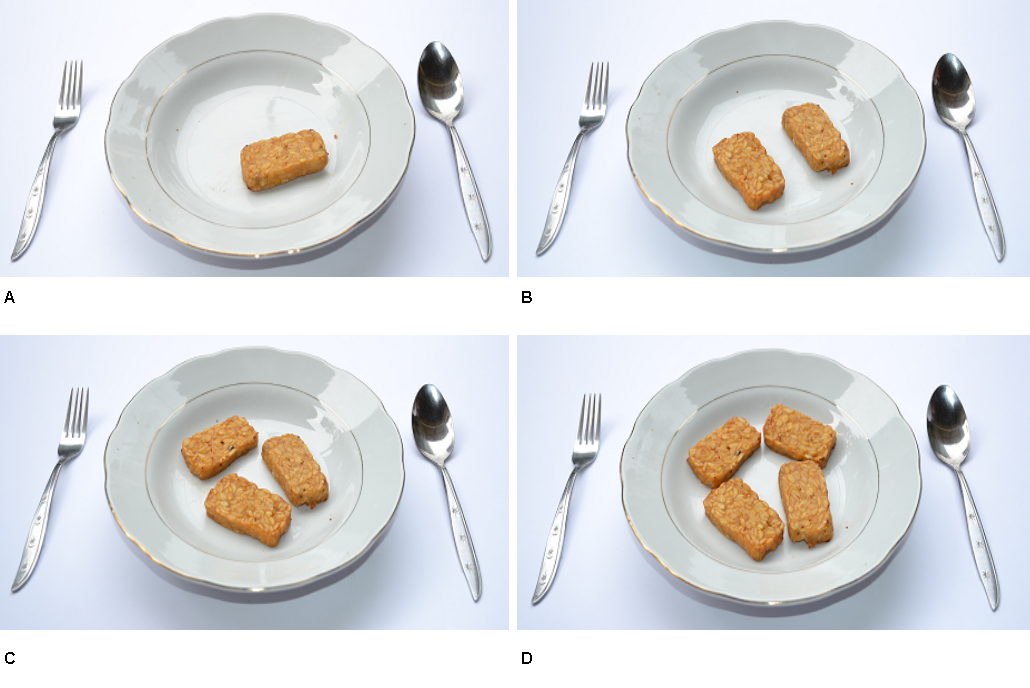


1. Rice


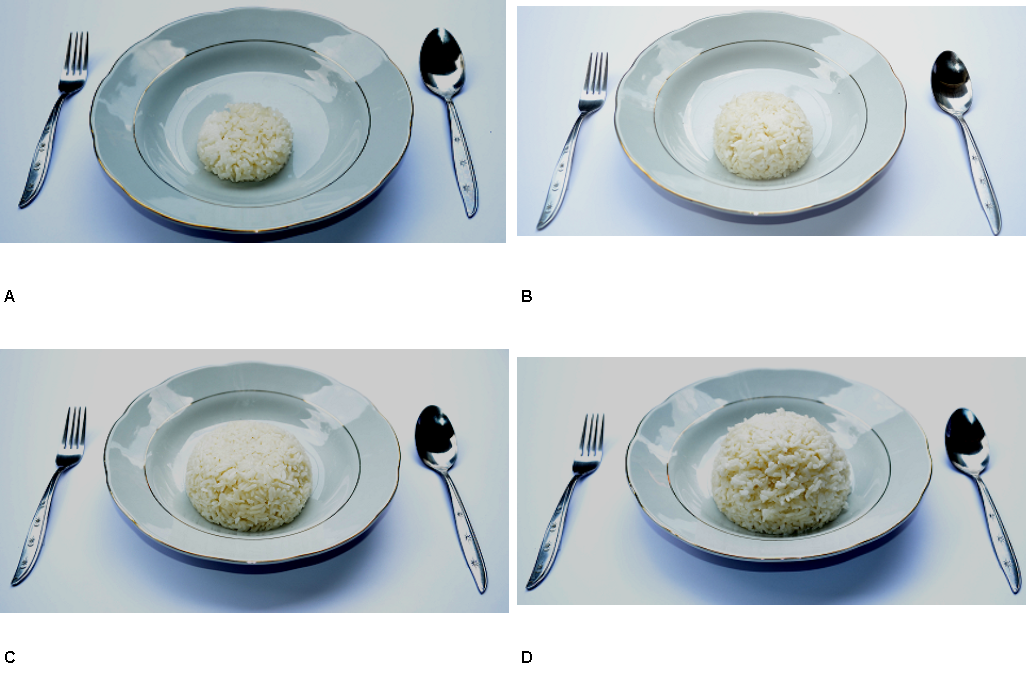


1. White bread


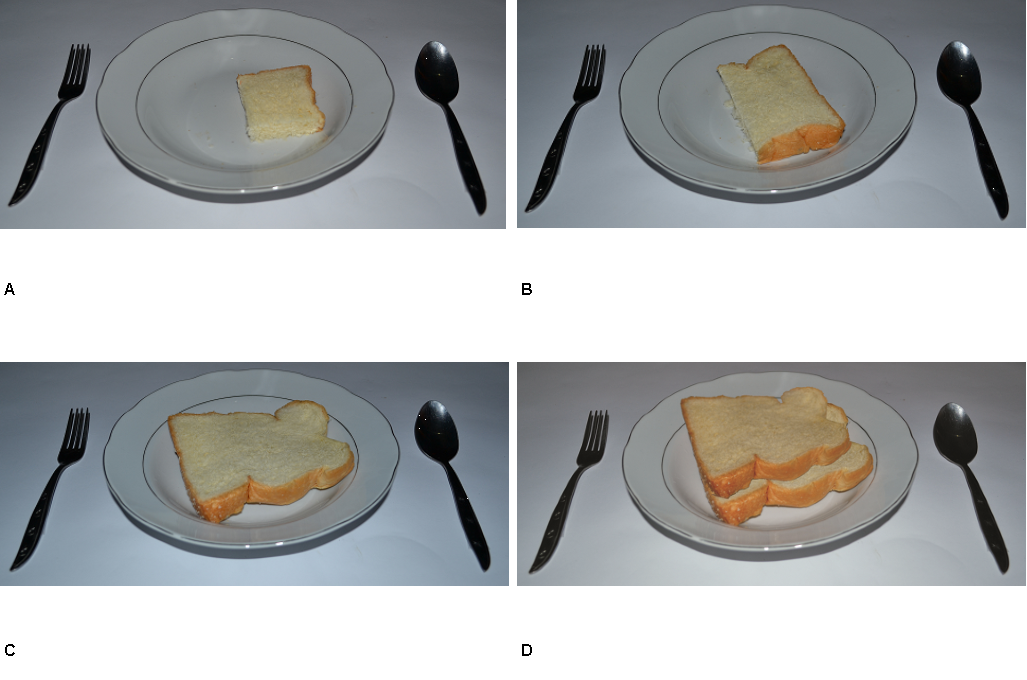


1. Pasta/noodle


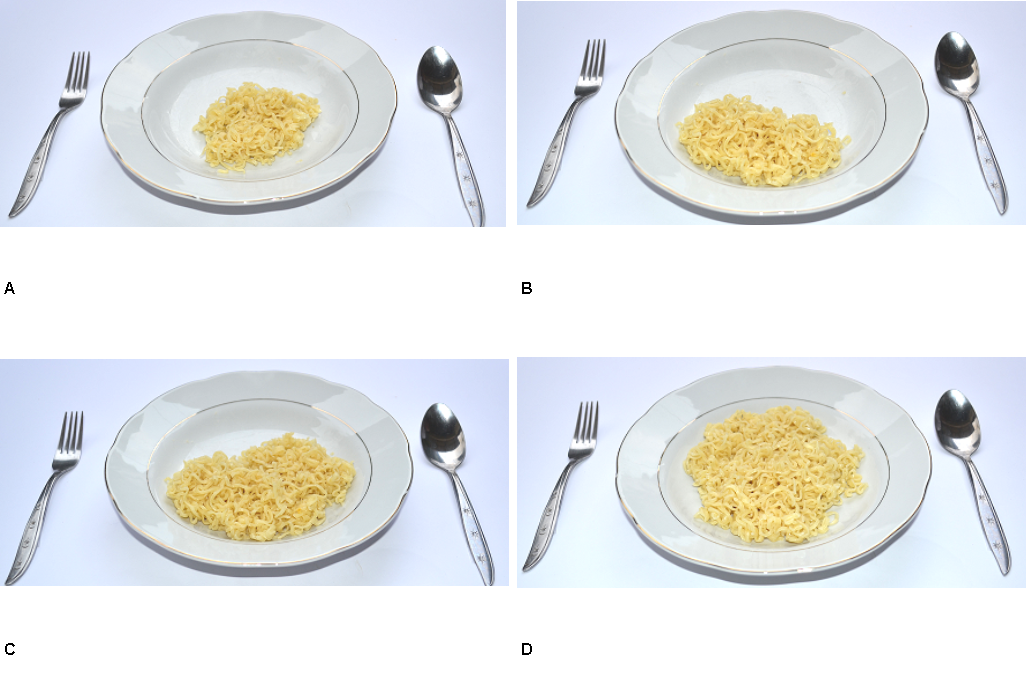


1.
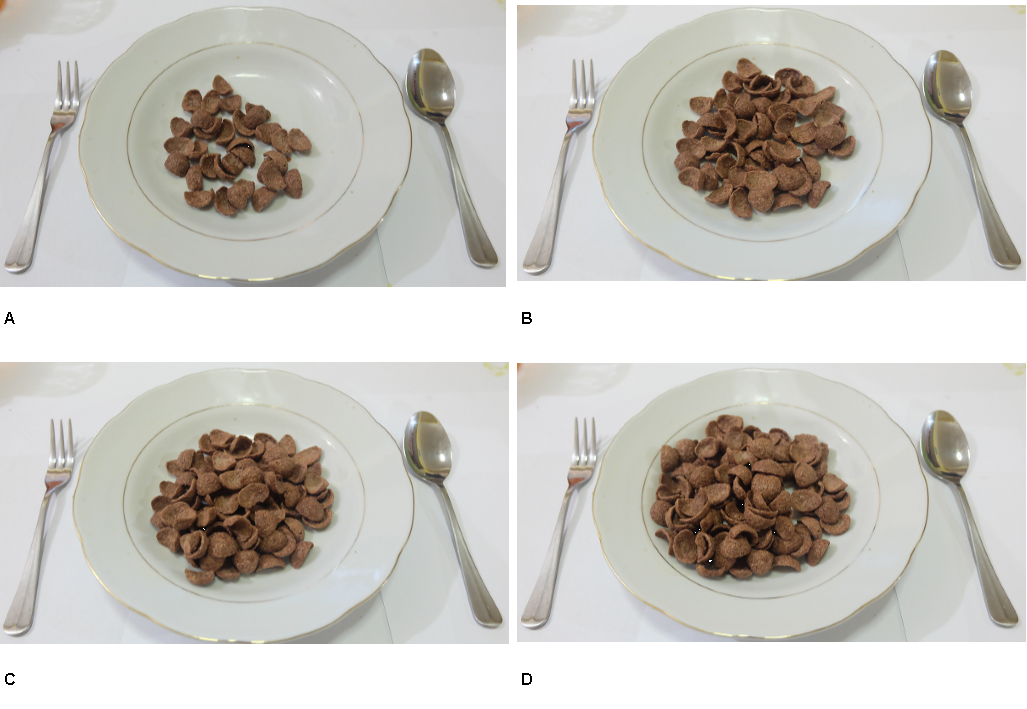
Breakfast cereal
2.
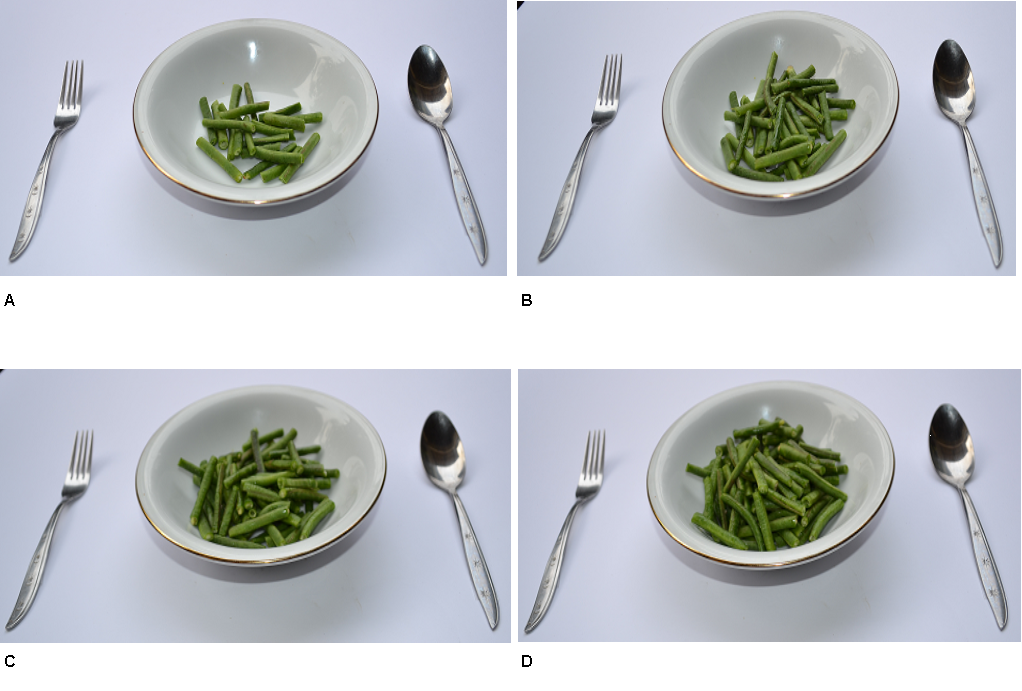
Long bean
3.
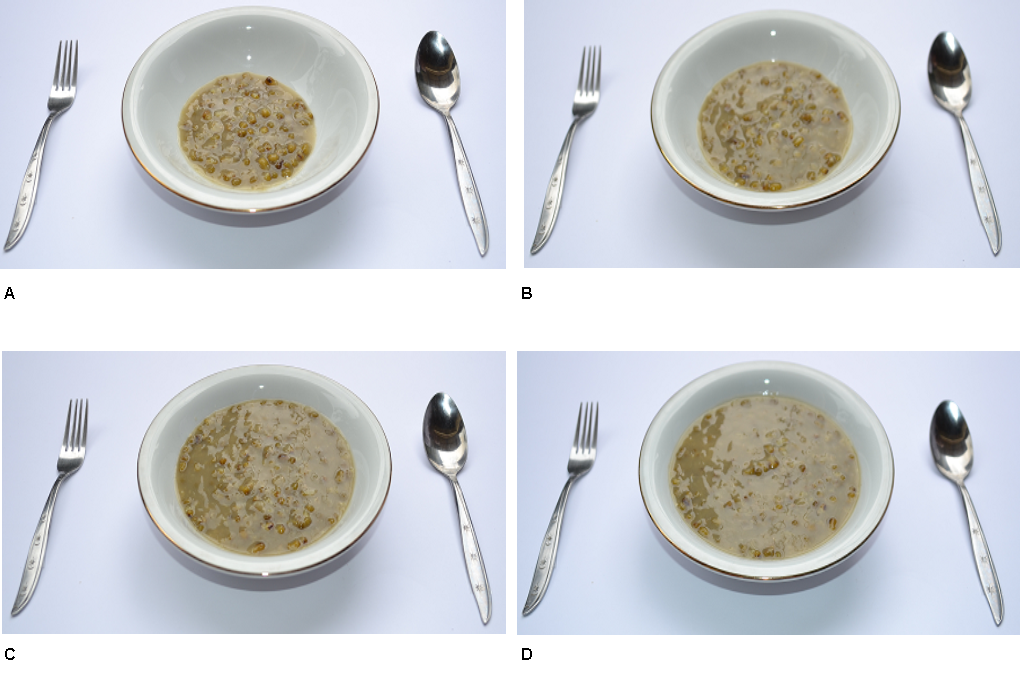
 Mung beans
4.
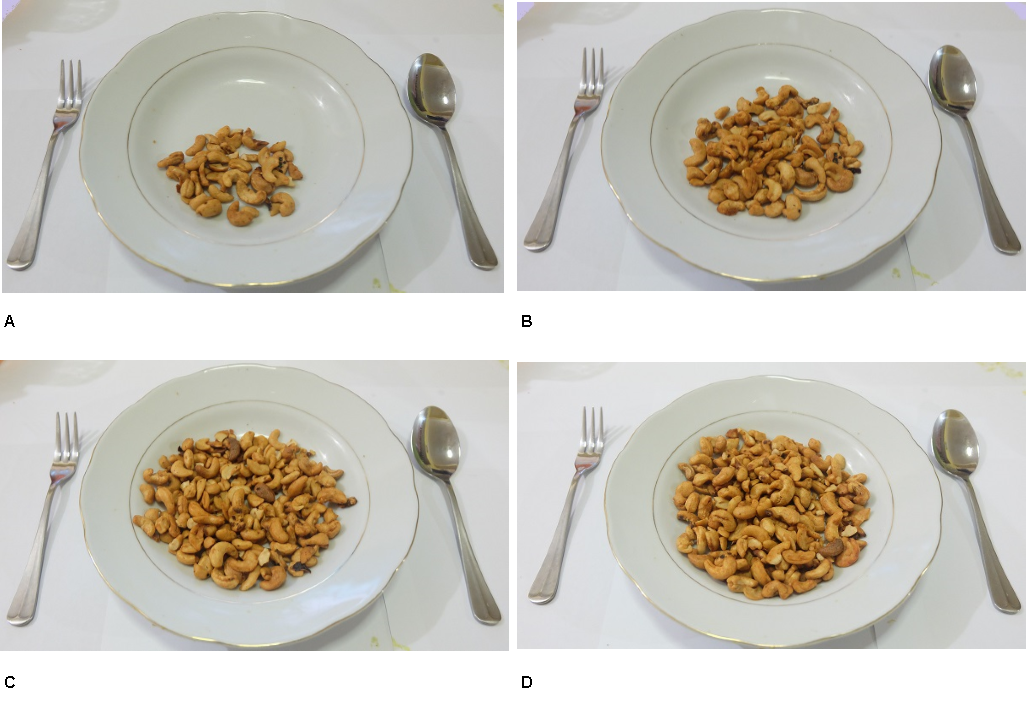
Peanuts
5. Corn


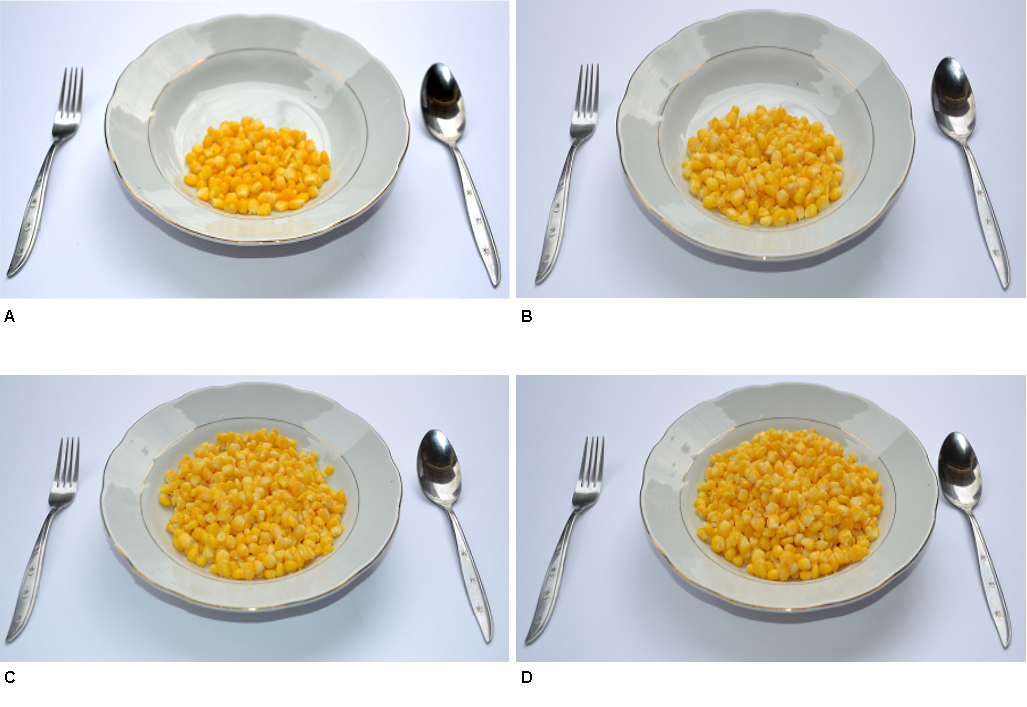


1. Potato


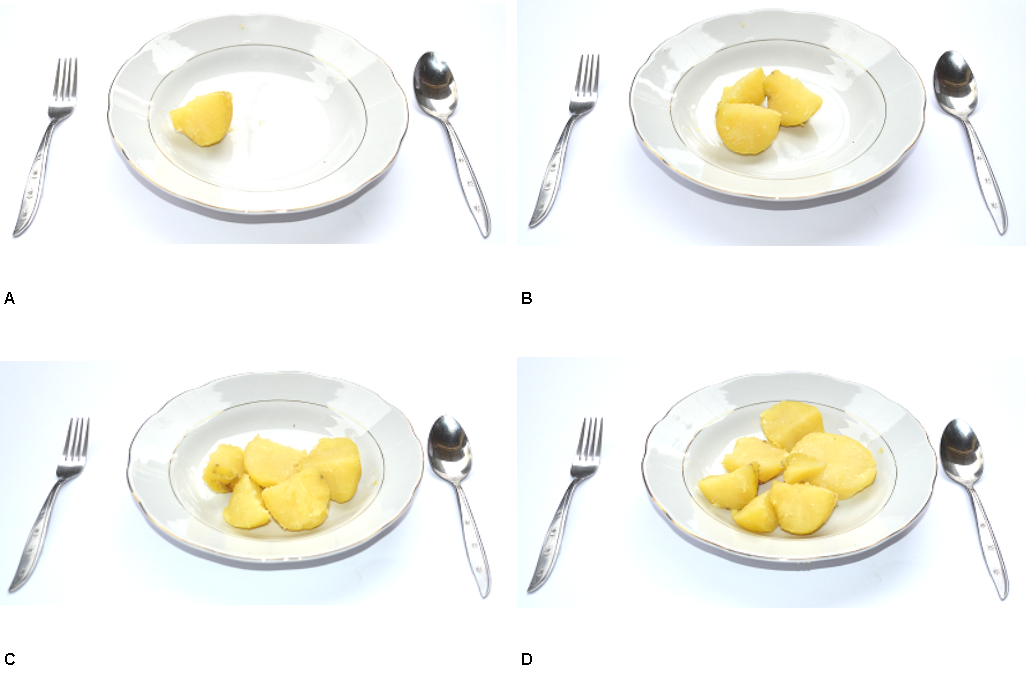


1.
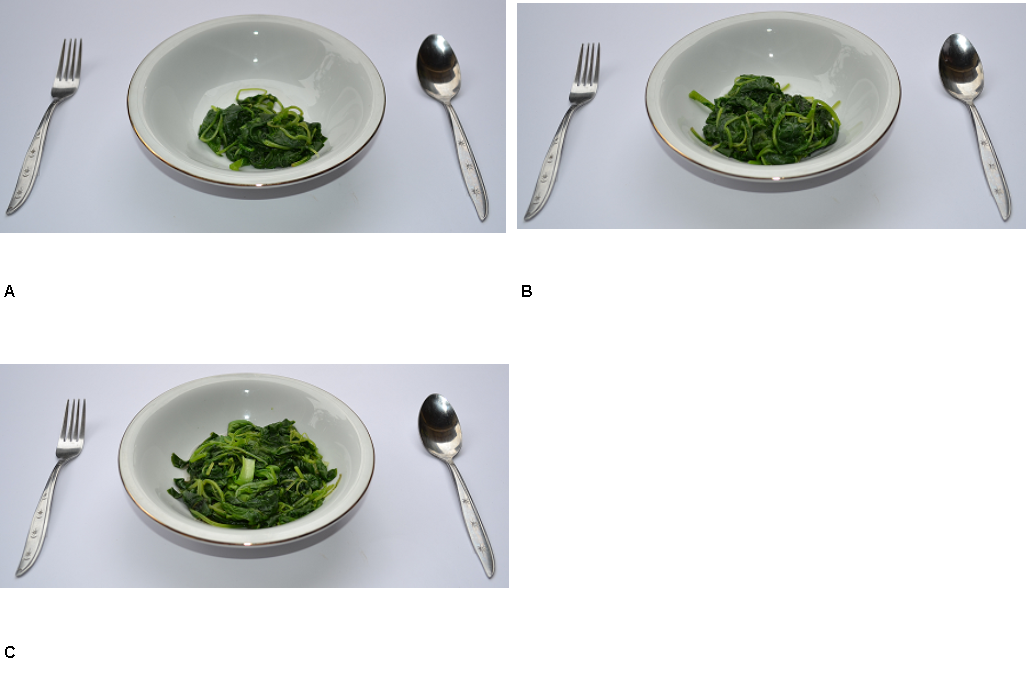
Spinach
2. Mushroom


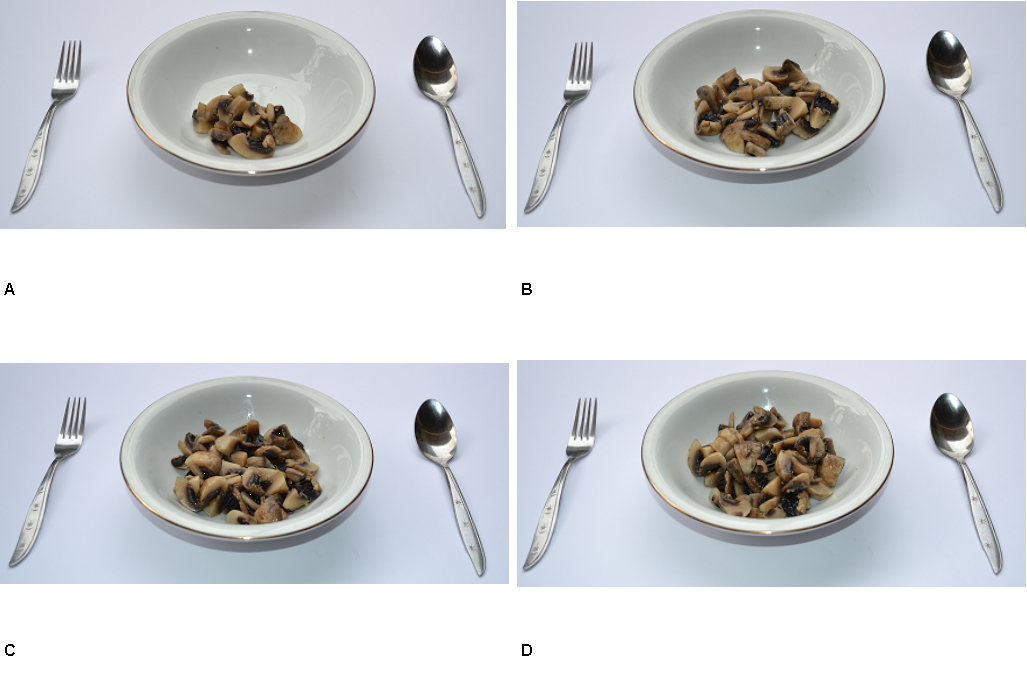


1.
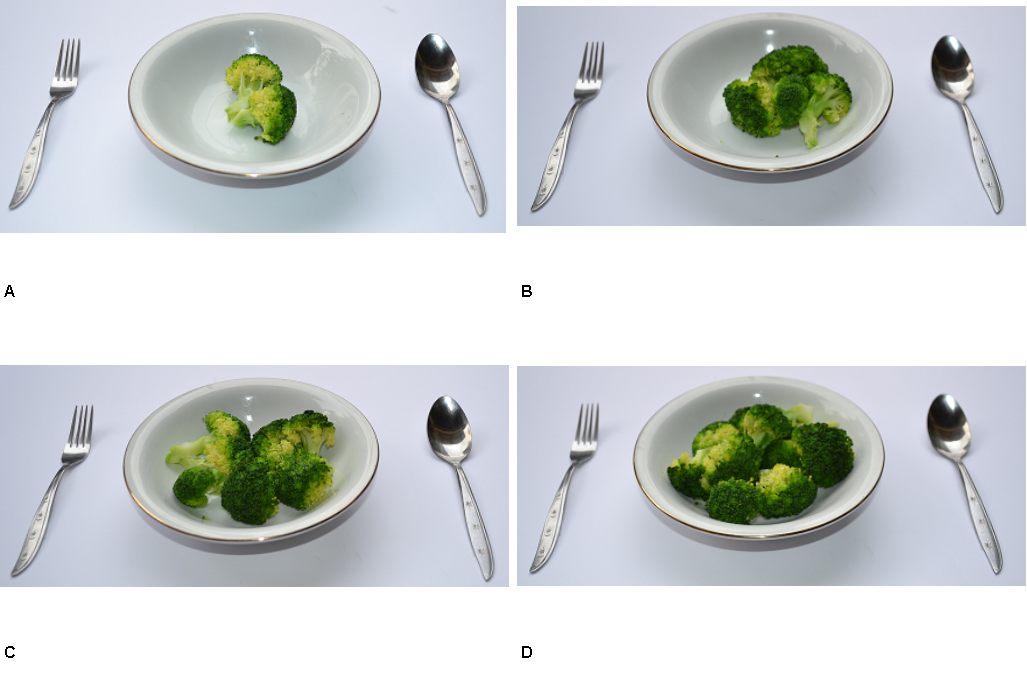
Broccoli
2.
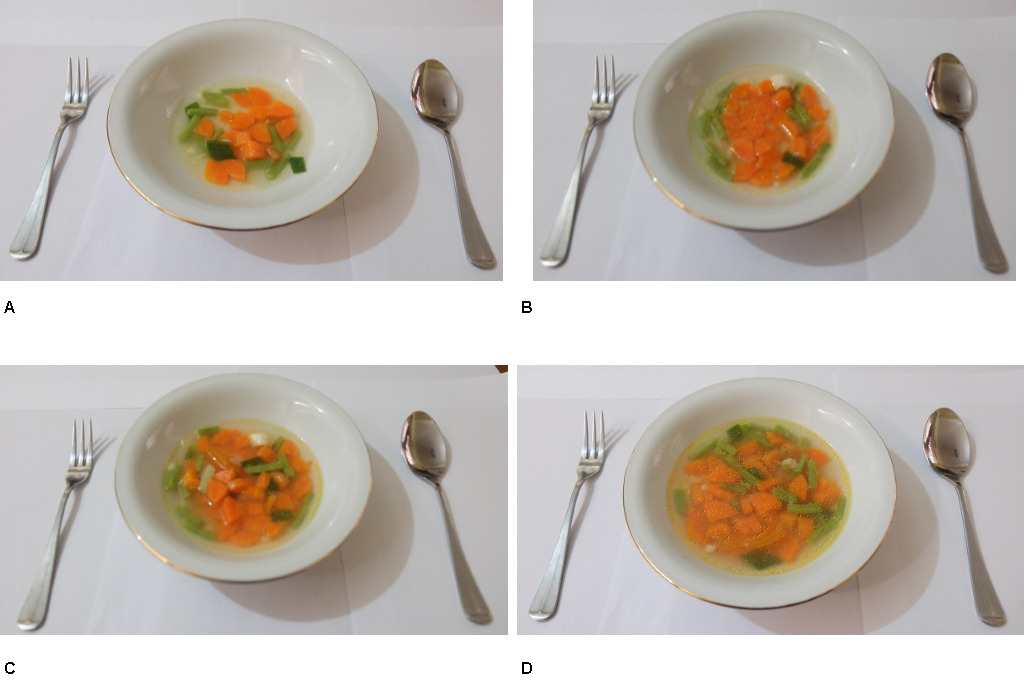
Other vegetables
3.
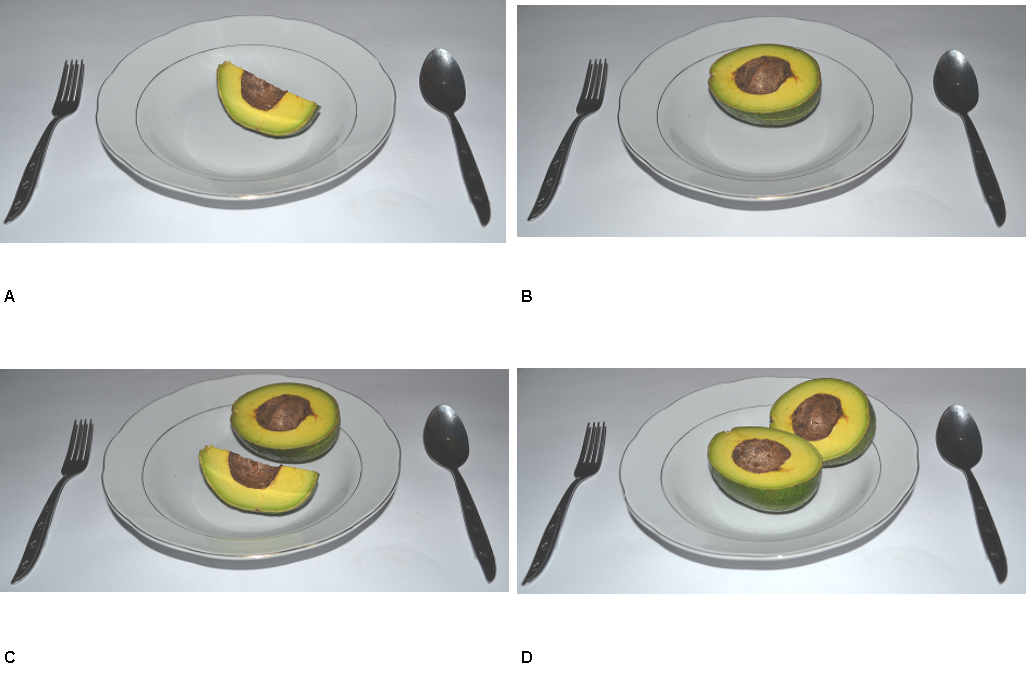
 Avocado
4. Milk (250 ml)


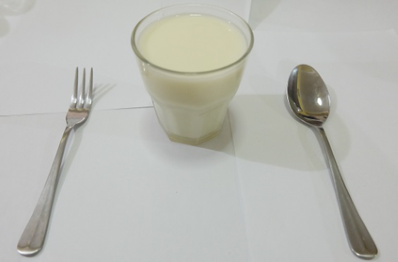


1. Cheese


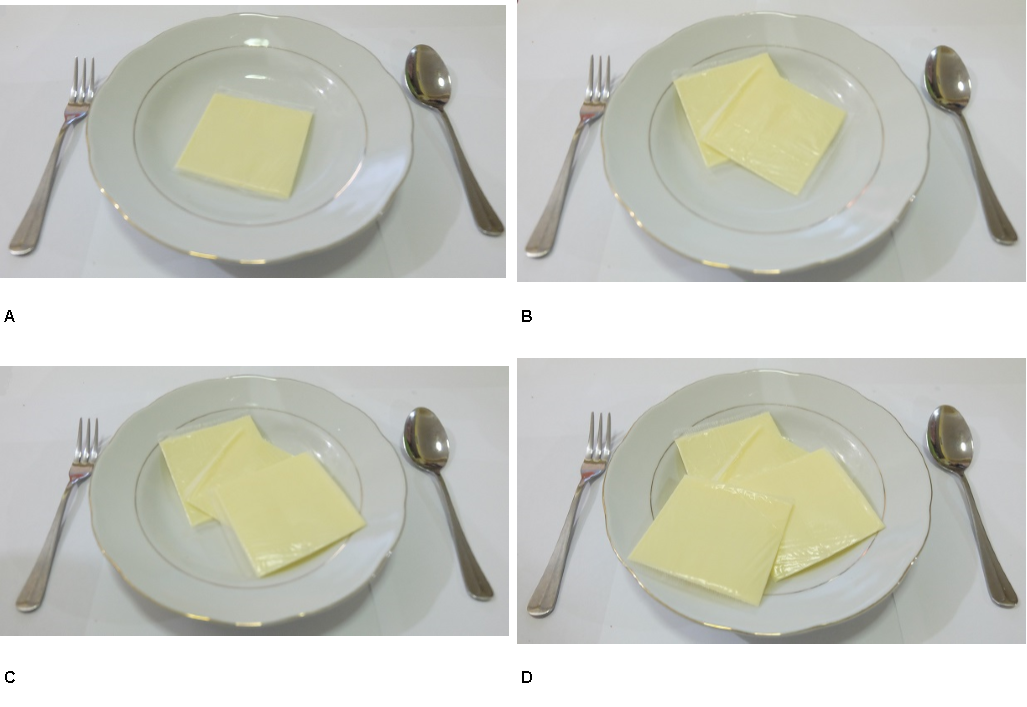


1. Yogurt


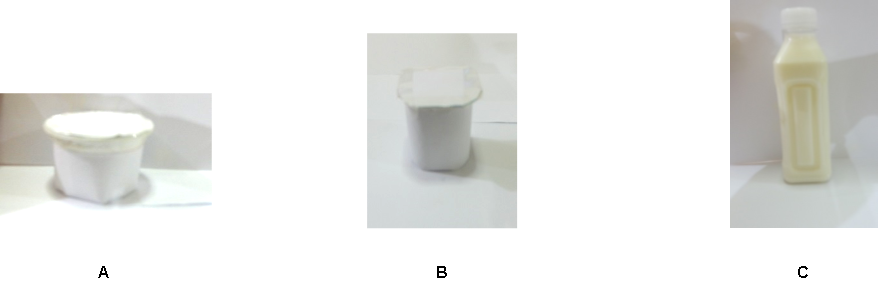

Supplement: Supplementary file 1 [file Datasheet1.docx]
